# UPF1 regulates the malignant biological behaviors of glioblastoma cells via enhancing the stability of Linc-00313

**DOI:** 10.1038/s41419-019-1845-1

**Published:** 2019-08-19

**Authors:** Lianqi Shao, Qianru He, Yunhui Liu, Xiaobai Liu, Jian Zheng, Jun Ma, Libo Liu, Han Li, Zhen Li, Yixue Xue

**Affiliations:** 1 Department of Neurobiology, College of Basic Medicine, China Medical University, Shenyang, 110122 People’s Republic of China; 2 Key Laboratory of Cell Biology, Ministry of Public Health of China, and Key Laboratory of Medical Cell Biology, Ministry of Education of China, China Medical University, Shenyang, 110122 People’s Republic of China; 3 Department of Neurosurgery, Shengjing Hospital of China Medical University, Shenyang, 110004 People’s Republic of China; 4 Liaoning Research Center for Clinical Medicine in Nervous System Disease, Shenyang, 110004 People’s Republic of China; 5 Key Laboratory of Neuro-oncology in Liaoning Province, Shenyang, 110004 People’s Republic of China

**Keywords:** Targeted therapies, Tumour biomarkers

## Abstract

There is growing evidence that the long non-coding RNAs(lncRNAs) play an important role in the biological behaviors of glioblastoma cells. In this study, we elucidated the function and possible effect and molecular mechanisms of lncRNA-Linc-00313 on the biological behaviors of glioblastoma cells as well as UPF1 function as a RNA-binding protein to enhance its stability. Here, we used qRT-PCR and western blot to measure the expression, cell Transfection to disrupt the expression of genes, cell viability analysis, quantization of apoptosis, cell migration, and invasion assays, Reporter vectors construction and luciferase assays to investigate the malignant biological behaviors of cells, human lncRNA microarrays, RNA-Immunoprecipitation, dual-luciferase gene reporter assay, half-life assay and chromatin immunoprecipitation to verify the binding sites, tumor xenograft implantation for in vivo experiment, SPSS 18.0 statistical software for data statistics. UPF1 and Linc-00313 were both upregulated in glioma tissues and cells. Knockdown of UPF1 or Linc-00313 significantly inhibited malignant biological behaviors of glioma cells by regulating miR-342-3p and miR-485-5p, which are downregulated and functioned as tumor suppressors in glioma. Furthermore, Linc-00313 could acted as a competing endogenous RNA(ceRNA) to regulate the expression of Zic4 by binding to miR-342-3p and miR-485-5p. Interestingly, Zic4 could bind to the promoters of UPF1 and Linc-00313 respectively and upregulate the expression of them. These results indicated that a positive-feedback loop was formed in the regulation of the biological behaviors of glioma cells. The study is the first to prove that the UPF1-Linc-00313-miR-342-3p/miR-485-5p-Zic4-SHCBP1 pathway forms a positive-feedback loop and regulates the biological behaviors of U87 and U251 cells, which might provide a new therapeutic target for glioma.

## Introduction

Glioblastoma is the most common primary malignant tumor of the central nervous system in adults with an annual incidence rate of approximately 6 per 100,000 persons^1^. Despite the combination of surgery, radiotherapy, and chemotherapy, the prognosis of glioblastoma remain poor due to their unique location and high degree of invasiveness^2^. Therefore, revealing the pathogenesis of glioblastoma and finding new biomarkers and therapeutic targets related to the tumorigenesis have become the focus of current research.

RNA-binding proteins are important for the regulation of gene expression at a post-transcriptional level^3^. RNA-binding proteins interact with RNA to regulate cell functions and are widely involved in RNA cleavage, transport, editing, intracellular localization, and translation regulation^4,5^. Long non-coding RNAs (LncRNAs) are a class of RNAs with transcripts longer than 200 nucleotides without protein-coding ability^6,7^, RNA-binding proteins are capable of binding to lncRNAs and directing the ribosomal protein complexes to the specific regions of lncRNAs. They can also regulate gene transcription as *cis*- or *trans*-acting factors by either directly affecting the sequences of the promoters or enhancers of nearby genes^8^, or by impacting on specific distant sites with the help of other accessory molecules^9^. RNA-binding proteins can specifically bind to lncRNAs and regulate its stability^10^, thereby regulating the development and progression of many diseases which including tumors.

UPF1 (RNA helicase and ATPase) is an RNA-binding protein, which is also an RNA helicase and a nucleic acid-dependent ATPase, and the ATPase activity is related to the 5′-3′ helicase activity^11^. Many UPF1 molecules accumulate in the 3′UTR regions of mRNAs which contain premature termination codons (PTCs)^12^. UPF1 is a key protein in the nonsense-mediated mRNA decay pathway^13^, which was reduced during the differentiation of neural stem cells into neurons^14^. Human lncRNA microarrays, catRAPID and RPISeq database were used to find that UPF1 can bind to the lncRNA-Linc-00313. Linc-00313 (also known as C21orf84 or CH507-42P11.5) is located on region 2 of the short arm of chromosome 21(21q22.3), whose transcript consists of 967 nucleotides. Linc-00313 was upregulated in lung cancer and used as a diagnostic biomarker for early stage lung cancer. Linc-00313 could promote the proliferation, migration, and invasion of lung cancer cells, and its upregulation is associated with poor prognosis in patients with lung cancer^15,16^. At present, the expression and functions of UPF1 and Linc-00313 in glioma remains uncharted.

Human miRNA microarrays and Starbase database reveals that Linc-00313 has binding sites with both miR-342-3p and miR-485-5p. MiR-342-3p is a single-stranded, non-coding microRNA that is 23 bases in length and downregulated in colon cancer^17^. MiR-342-3p was also downregulated in gliomas^18^, which could inhibit the proliferation and invasion of glioma cells^19^. MiR-485-5p is located on human chromosome 14. Studies have shown that miR-485-5p was downregulated in glioma tissues and cell lines. The overexpression of miR-485-5p could inhibit the proliferation, migration, and invasion of glioma cells^20^. LncRNA can affect the biological behaviors of tumor cells through the ceRNA mechanism, that is, lncRNA competes with target mRNA for miRNA binding, or lncRNA can act as a “miRNA sponge”, which indirectly inhibits miRNA from regulating their target mRNA^21^. There is no published report on the functions of Linc-00313, miR-342-3p, and miR-485-5p in glioma tissues and cells.

The transcription factor Zic4 is one of the members of ZIC family of C2H2-type zinc finger proteins and plays an important role in the developmental process. In previous research we used miRanda database to find that miR-342-3p and miR-485-5p can bind to the 3′UTR region of Zic4, respectively. The detection rates of serum Zic4 antibodies in patients with tumor-related neurological diseases and small cell lung cancer were significantly higher than that in healthy individuals^22^. Zic4 was upregulated in medulloblastoma tissue^23^. Otherwise, JASPAR CORE database showed that Zic4 can bind to the promoter regions of SHCBP1. SHCBP1 is a member of the Src homolog and collagen homolog (Shc) protein family. It is a regulatory protein for cell receptors that can activate growth factor receptor-mediated signaling pathways. Studies have shown that SHCBP1 was upregulated in breast cancer tissues and cells and could promote breast cancer cell proliferation and inhibit apoptosis^24^. SHCBP1 is also upregulated in liver cancer tissues and acts as a tumor-promoting gene by activating the ERK signaling pathway^25^.

In this study, the endogenous expression of UPF1, Linc-00313, miR-342-3p, miR-485-5p, Zic4, and SHCBP1 in glioma tissues and cells were determined. The regulatory relationships among these factors and their effects on the biological behaviors of glioma cells were further investigated. The objective of the study was to provide new theoretical and experimental evidence on brain glioma tumorigenesis and development, and to identify new targets for the treatment of gliomas.

## Results

### UPF1 and Linc-00313 were upregulated in glioma tissues and cells, knockdown of UPF1, Linc-00313 inhibited the malignant biological behaviors of glioma cells

**Fig. 1:**
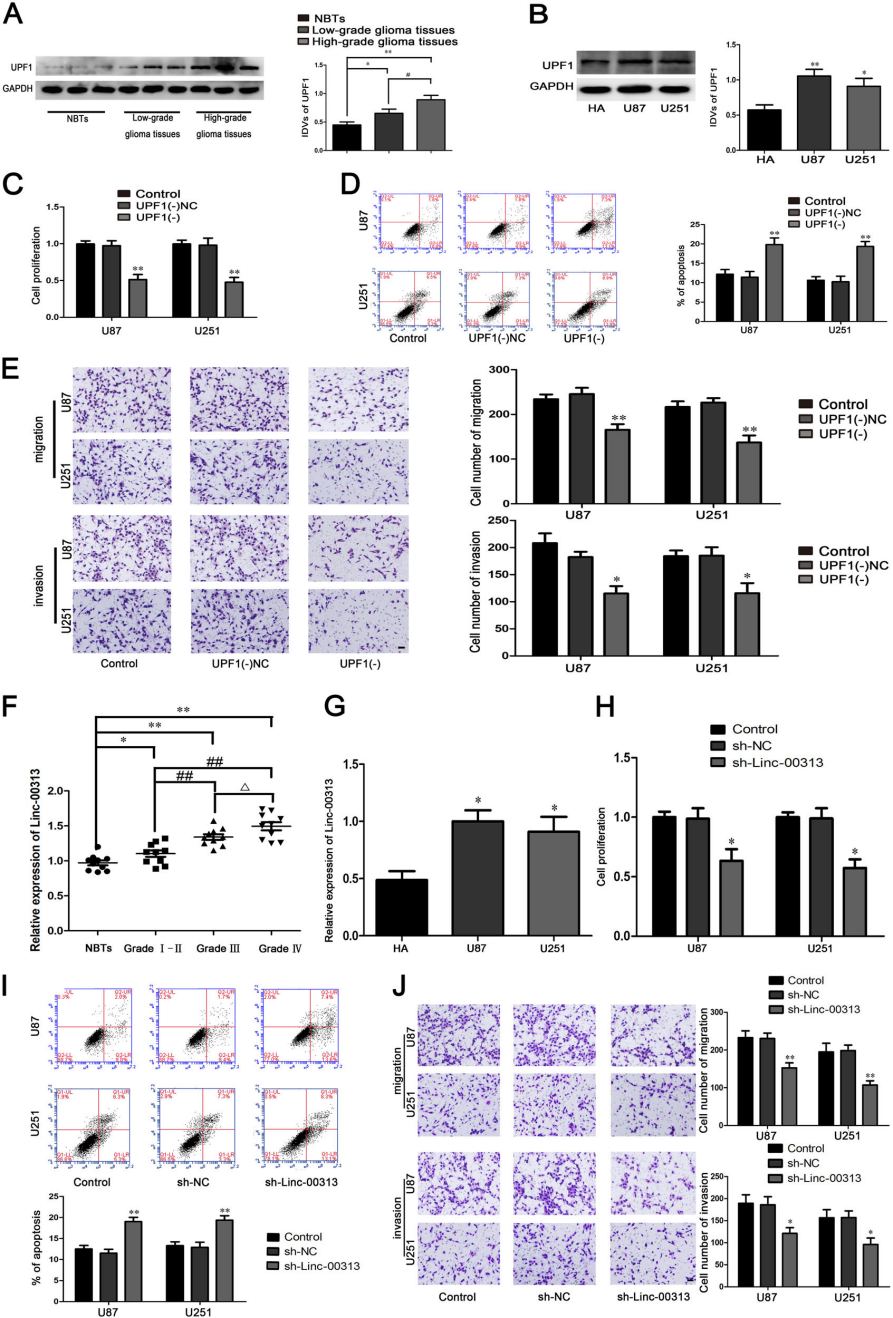
The expression and effect of UPF1 and Linc-00313 in glioma. **a** The UPF1 protein expression levels in normal brain tissues (NBTs), and glioma tissues of different grades. Data are presented as the mean ± SD (*n* = 9, each group). **P < 0.01 vs. NBTs group; ^#^*P* < 0.05 vs^.^ low-grade glioma tissues group. **b** The expression of UPF1 in human astrocytes (HA) and glioblastoma cell lines (U87 and U251). **c** Cell Counting Kit-8 (CCK-8) assay was used to measure the effect of UPF1 on the proliferation of U87 and U251. **d** The apoptotic percentages of U87 and U251 after UPF1 knockdown. **e** Transwell assays was used to measure the effect of UPF1 on cell migration and invasion of U87 and U251 after UPF1 knockdown. Data are presented as the mean ± SD (*n* = 3, each group). **P < 0.01 vs. UPF1(−)NC group. Scale bars represent 40 μm. **f** The Linc-00313 expression levels in normal brain tissues (NBTs), and glioma tissues of different grades. Data are presented as the mean ± SD (*n* = 10, each group). ^ΔΔ^*P* < 0.01 vs. NBTs group; **P < 0.01 vs. Grade I–II group; ^#^*P* < 0.05 vs. Grade III group. **g** The expression of Linc−00313 in human astrocytes (HA) and glioblastoma cell lines (U87 and U251). Data are presented as the mean ± SD. **h** Cell Counting Kit-8 (CCK-8) assay was used to measure the effect of Linc-00313 on the proliferation of U87 and U251. **i** The apoptotic percentages of U87 and U251 after Linc-00313 knockdown. **j** Transwell assays was used to measure the effect of Linc-00313 on cell migration and invasion of U87 and U251 after Linc-00313 knockdown. Data are presented as the mean ± SD (*n* = 3, each group). **P < 0.01 vs. sh-NC group. Scale bars represent 40 μm

As shown in Fig. 1a, b, the expression of UPF1 in glioma tissues and U87, U251 cells was significantly higher than that in normal brain tissues and HA cells. To explore the function of UPF1 in U87 and U251 cells, the biological behaviors of U87 and U251 cells were detected. The proliferation, migration, and invasion of U87 and U251 cells were significantly decreased and the apoptosis were significantly increased in the UPF1(−) group compared with the UPF1(−)NC group (Fig. 1c–e).

The expression of Linc-00313 in glioma tissues and U87, U251 cells was significantly higher than that in normal brain tissues and HA cells (Fig. 1f, g). The proliferation, migration, and invasion of U87 and U251 cells were significantly decreased and the apoptosis were significantly increased in the sh-Linc-00313 group compared with the sh-NC group (Fig. 1h–j).

### MiR-342-3p and miR-485-5p were downregulated in glioma tissues and cells, and functioned as tumor suppressors in U87 and U251 cells

**Fig. 2:**
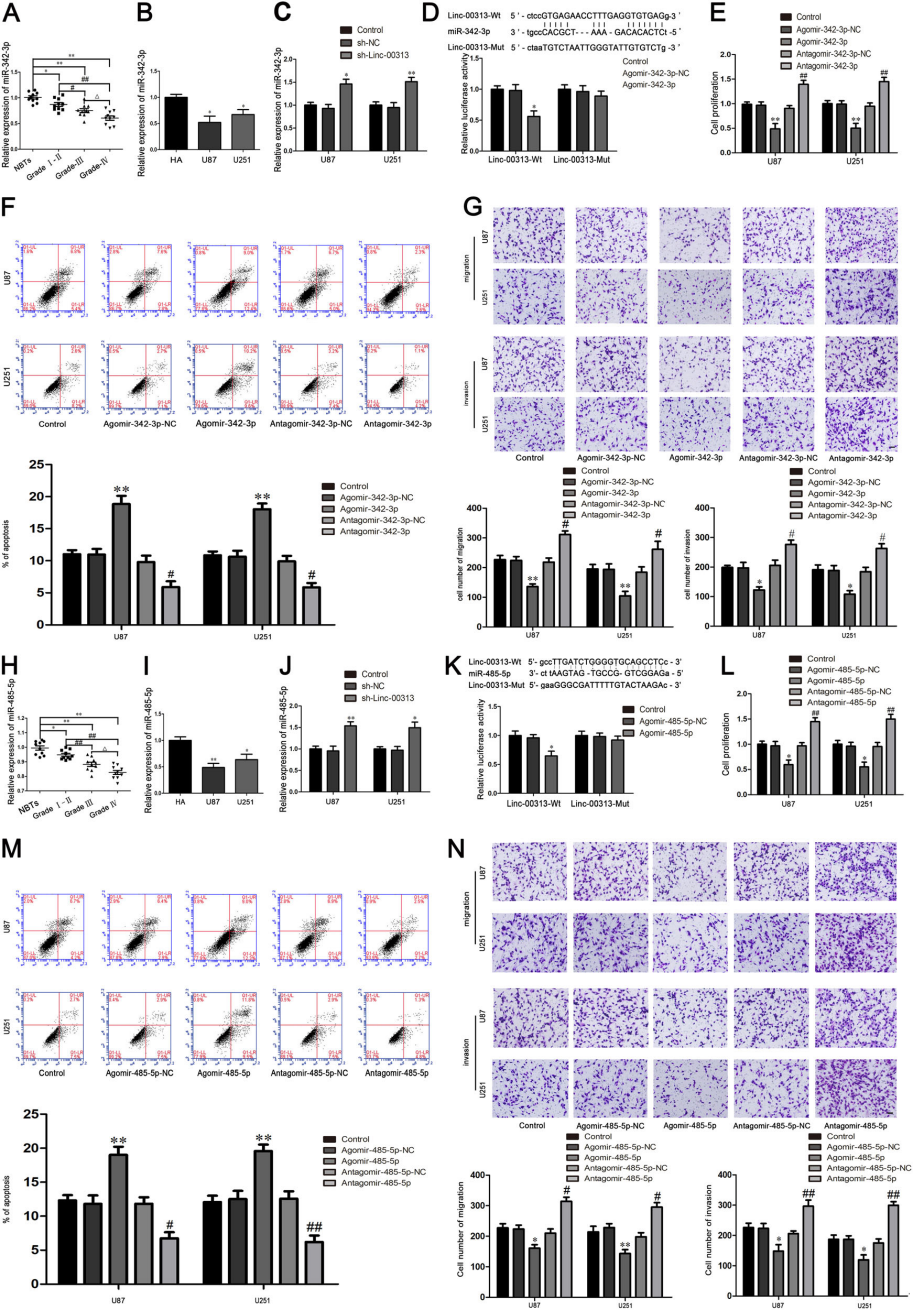
The expression and effect of miR-342-3p and miR-485-5p in glioma. **a** The miR-342-3p expression levels in normal brain tissues (NBTs), and glioma tissues of different grades. Data are presented as the mean ± SD (*n* = 10, each group). ^ΔΔ^*P* *<* 0.01 vs. NBTs group; **P < 0.01 vs. Grade I–II group; ^#^*P* < 0.05 vs. Grade III group. **b** The expression of miR-342-3p in human astrocytes (HA) and glioblastoma cell lines (U87 and U251). **c** The expression of miR-342-3p after Linc-00313 knockdown in U87 and U251 cells. **d** The predicted miR-342-3p binding site in the Linc-00313 sequence (Linc-00313-Wt) and the designed mutant sequence of miR-342-3p binding site (Linc-00313-Mut) are indicated. Relative luciferase activity was conducted after cells were transfected with Linc-00313-Wt or Linc-00313-Mut. Data were presented as the mean ± SD (*n* = 3, each group). **P < 0.01 vs. Linc-00313Wt + Agomir-342-3p-NC. **e** Cell Counting Kit-8(CCK-8) assay was used to measure the effect of miR-342-3p on the proliferation of U87 and U251. **f** The apoptotic percentages of U87 and U251 cells were detected after miR-342-3p overexpression or inhibition. **g** Transwell assays was used to measure the effect of miR-342-3p on the migration and invasion of U87 and U251 cells. Scale bars represent 40 μm. Data are presented as the mean ± SD (*n* = 3, each group). *P < 0.05 vs. Agomir-342-3p-NC group; ^##^*P* < 0.01 vs. Antagomir-342-3p-NC group. **h** The miR-485-5p expression levels in normal brain tissues (NBTs), and glioma tissues of different grades. Data are presented as the mean ± SD (*n* = 10, each group). ^ΔΔ^*P* < 0.01 vs. NBTs group; **P < 0.01 vs. Grade I–II group; ^#^*P* < 0.05 vs^.^ Grade III group. **i** The expression of miR-485-5p in human astrocytes (HA) and glioblastoma cell lines (U87 and U251). **j** The expression of miR-485-5p after Linc-00313 knockdown in U87 and U251 cells. **k** The predicted miR-485-5p binding site in the Linc-00313 sequence(Linc-00313-Wt) and the designed mutant sequence of miR-485-5p binding site (Linc-00313-Mut) are indicated. Relative luciferase activity was conducted after cells were transfected with Linc-00313-Wt or Linc-00313-Mut. Data were presented as the mean ± SD (*n* = 3, each group). **P < 0.01 vs. Linc-00313Wt + Agomir-485-5p-NC. **l** Cell Counting Kit-8 (CCK-8) assay was used to measure the effect of miR-485-5p on the proliferation of U87 and U251. **m** The apoptotic percentages of U87 and U251 cells were detected after miR-485-5p overexpression or inhibition. **n** Transwell assays was used to measure the effect of miR-485-5p on the migration and invasion of U87 and U251 cells. Scale bars represent 40 μm. Data are presented as the mean ± SD (*n* = 3, each group). *P < 0.05 vs. Agomir-485-5p-NC group; ^##^*P* < 0.01 vs. Antagomir-485-5p-NC group

Compared with normal brain tissues and HA cells the expression of miR-342-3p and miR-485-5p were significantly decreased in human glioma tissues and U87, U251 cells (Fig. 2a, b, h, i). As shown in Fig. 2c, j, the expression of miR-342-3p and miR-485-5p were significantly increased in sh-Linc-00313 group compared with the sh-NC group. Luciferase reporter assay was applied to elucidate the molecular mechanisms. The luciferase activities of Linc-00313-Wt + Agomir-342-3p group (Fig. 2d) and Linc-00313-Wt + Agomir-485-5p group (Fig. 2k) were significantly decreased compared with Linc-00313-Wt + Agomir-342-3p-NC group (Fig. 2d) and Linc-00313-Wt + Agomir-485-5p-NC group (Fig. 2k).

The proliferation, migration, and invasion of U87 and U251 cells were significantly decreased and the apoptosis were significantly increased in the Agomir-342-3p group and Agomir-485-5p group compared with the Agomir-342-3p-NC group and Agomir-485-5p-NC group, the Antagomir-342-3p group and Antagomir-485-5p group had the opposite effect (Fig. 2e–g, l–n).

### UPF1 promoted the stability of Linc-00313 and regulated the biological behaviors of glioma cells

**Fig. 3:**
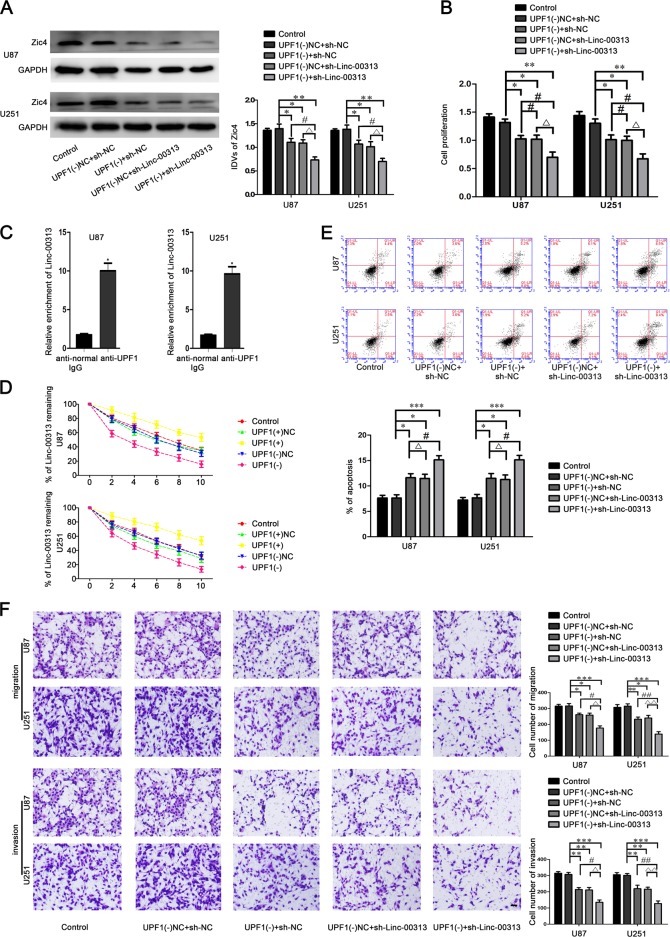
UPF1 binds Linc-00313 and enhances the effect of Linc-00313 on glioma. **a** Western blot assay were used to detect the Zic4 expression regulated by UPF1 and Linc-00313. **b** CCK-8 assay was used to measure the proliferation of U87 and U251 regulated by UPF1 and Linc-00313. **c** RNA IP assay was used to detect the binding between UPF1 and Linc-00313. **d** Linc-00313 RNA half-life measured by qRT-PCR after actinomycin D treatment. **e** Flow cytometry analysis to evaluate the effect of UPF1 and Linc-00313 on the apoptosis of U87 and U251 cells. **f** Transwell assays was used to measure the effect of UPF1 and Linc-00313 on the migration and invasion of U87 and U251 cells. Data are presented as the mean ± SD (*n* = 3, each group). *P < 0.05 vs. UPF1(−)NC + sh-NC

Microarrays were used to Screen the UPF1-related lncRNAs. We found that Linc-00313 has the lowest relative expression after downregulation of UPF1 (Fig. S3A). The binding sites of UPF1 and Linc-00313 were predicted by catRAPID and RPISeq database (Fig. S1A). Then we further verified the binding sites between UPF1 and linc-00313 through the RIP experiment.The relative enrichment levels of Linc-00313 was increased in the anti-UPF1 group than that in the anti-normal group (Fig. 3c). As shown in Fig. 3d, the half-life of Linc-00313 in UPF1(+) group increased, The half-life of Linc-00313 in UPF1(−) group decreased. These results indicated that UPF1 could delay the degradation of Linc-00313. The binding sites of Linc-00313 and miR-342-3p, miR-485-5p were predicted by starbase database (Fig. S1B). The expression of Zic4 and cell proliferation, migration, and invasion in the UPF1(−) + sh-NC group, UPF1(−)NC + sh-Linc-00313 group, UPF1(−) + sh-Linc-00313 group were significantly decreased compared with the UPF1(−)NC + sh-NC group. And the apoptosis were significantly increased (Fig. 3a–f).

### Linc-00313 promoted the malignant biological behaviors of glioma cells by regulating miR-342-3p and miR-485-5p

**Fig. 4:**
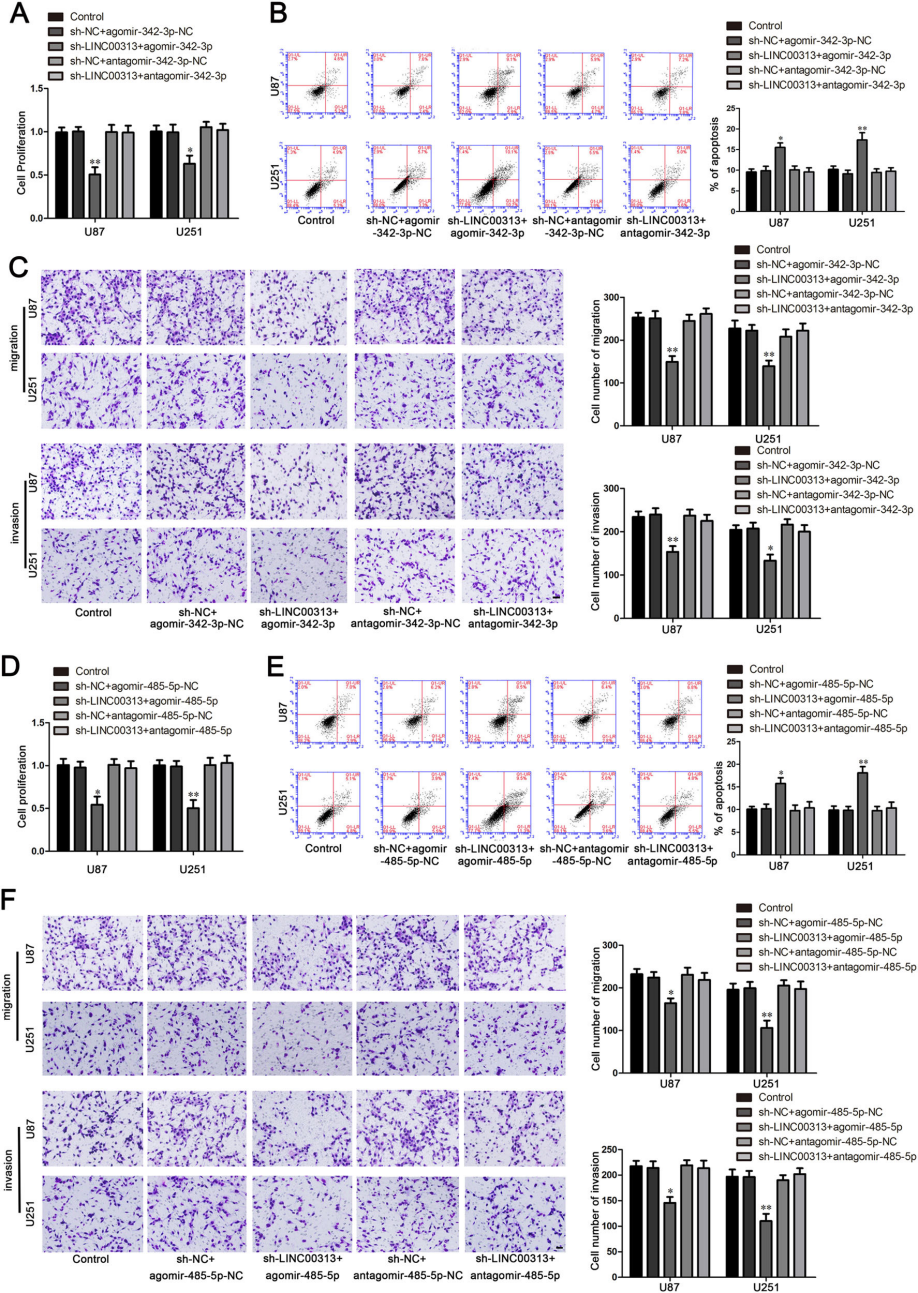
MiR-342-3p and miR-485-5p mediated the tumor-suppressive effects of Linc-00313 knockdown on glioma. **A** CCK-8 assay was used to measure the effect of Linc-00313 and miR-342-3p on the proliferation of U87 and U251 cells. **b** Flow cytometry analysis to evaluate the effect of Linc-00313 and miR-342-3p on the apoptosis of U87 and U251 cells. **c** Transwell assays was used to measure the effect of Linc-00313 and miR-342-3p on the migration and invasion of U87 and U251 cells. Data are presented as the mean ± SD (*n* = 3, each group). **P < 0.01 vs. sh-NC + agomir-342-3p-NC. **d** CCK-8 assay was used to measure the effect of Linc-00313 and miR-485-5p on the proliferation of U87 and U251 cells. **e** Flow cytometry analysis to evaluate the effect of Linc-00313 and miR-485-5p on the apoptosis of U87 and U251 cells. **f** Transwell assays was used to measure the effect of Linc-00313 and miR-485-5p on the migration and invasion of U87 and U251 cells. Scale bars represent 40 μm. Data are presented as the mean ± SD (*n* = 3, each group). **P < 0.01 vs. sh-NC + agomir-485-5p-NC

To determine the effects of Linc-00313, miR-342-3p, and miR-485-5p on U87 and U251 cells, the stable sh-Linc-00313 cells were transfected with miR-342-3p or miR-485-5p agomir and antagomir. And we found that the cell proliferation, migration, and invasion in the sh-Linc-00313 + agomir-342-3p group were significantly decreased compared with the sh-NC + agomir-342-3p-NC group. And the apoptosis were significantly increased. The sh-Linc-00313 + agomir-485-5p group had the same effect (Fig. 4a–f).

### Zic4 promoted the transcription of UPF1, Linc-00313, and the malignant biological behaviors of glioma cells

**Fig. 5:**
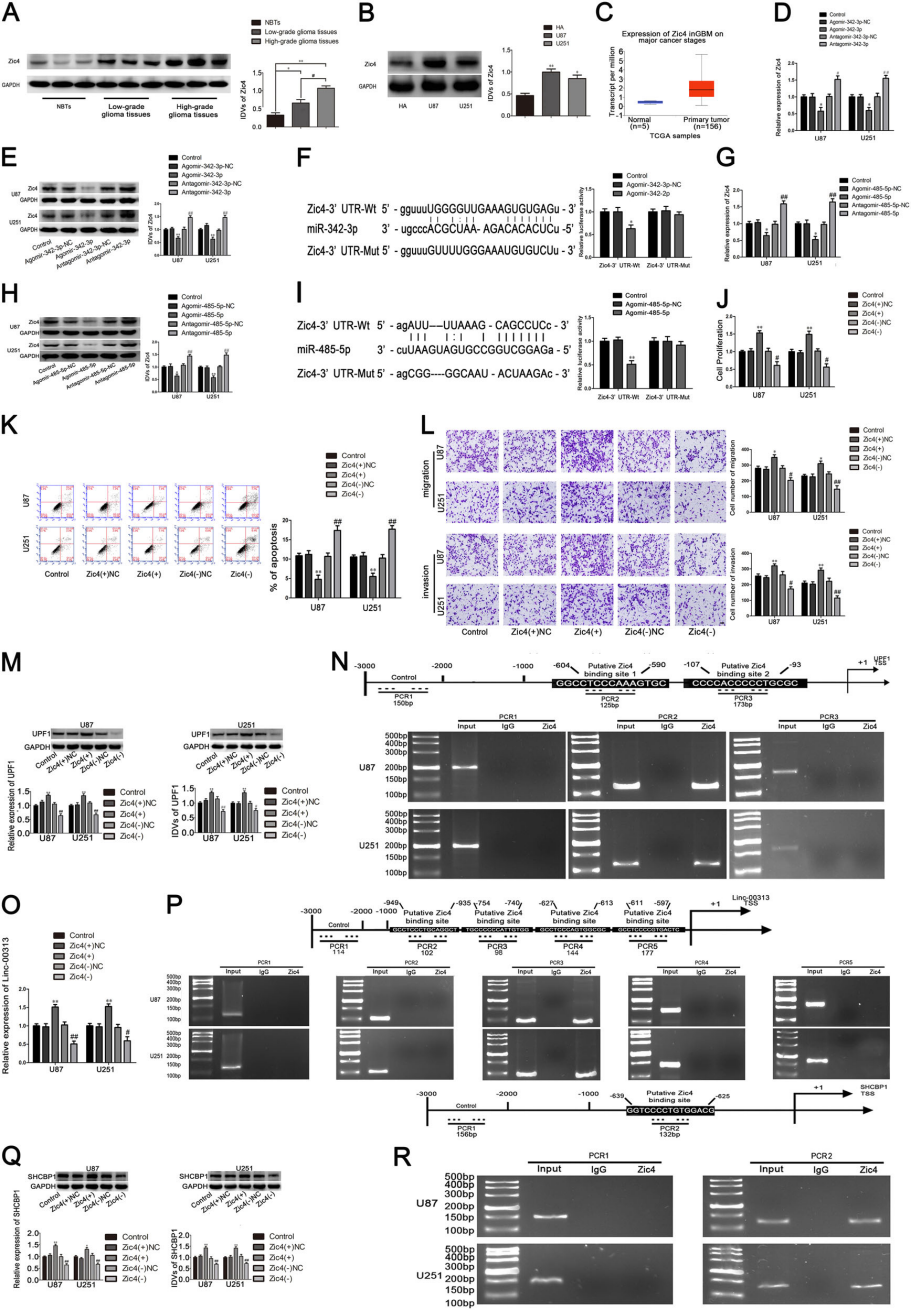
Zic4 endogenous expression and its effect on the proliferation, migration, invasion, and apoptosis in U87 and U251, and the expression of UPF1, Linc-00313 and SHCBP1, as well as the Zic4 expression regulated by miR-342-3p and miR-485-5p. **a** The Zic4 protein expression levels in normal brain tissues(NBTs), and glioma tissues of different grades. Data are presented as the mean ± SD (*n* = 9, each group). **P < 0.01 vs. NBTs group; ^#^*P* < 0.05 vs. low-grade glioma tissues group. **b** The expression level of Zic4 in human astrocytes (HA) and glioblastoma cell lines (U87 and U251). **c** The expression level of Zic4 in TCGA database. **d** Real-time PCR and **e** Western blot assay were used to detect the Zic4 expression after miR-342-3p overexpression or knockdown. **f** The predicted miR-342-3p binding sites in the 3′UTR region of Zic4 (Zic4-3′UTR-Wt) and the designed mutant sequence (Zic4-3′UTR-Mut) are indicated. Relative luciferase activity was conducted after cells were transfected with Zic4-3′UTR-Wt or Zic4-3′UTR-Mut. Data were presented as the mean ± SD (*n* = 3, each group). **P < 0.01 vs. Zic4-3′UTR-Wt + Agomir-342-3p-NC group. **g** Real-time PCR and **h** Western blot assay were used to detect the Zic4 expression after miR-485-5p overexpression or knockdown. **i** The predicted miR-485-5p binding sites in the 3′UTR region of Zic4 (Zic4-3′UTR-Wt) and the designed mutant sequence (Zic4-3′UTR-Mut) are indicated. Data were presented as the mean ± SD (*n* = 3, each group). **P < 0.01 vs. Zic4-3′UTR-Wt + Agomir-485-5p-NC group. **j** CCK-8 assay was used to measure the effect of Zic4 on the proliferation of U87 and U251 cells. **k** The apoptotic percentages of U87 and U251 were detected after Zic4 overexpression or knockdown. **l** Transwell assays was used to measure the effect of Zic4 on cell migration and invasion of U87 and U251 cells. Scale bars represent 40 μm. Data are presented as the mean ± SD (*n* = 3, each group). **P < 0.01 vs. Zic4(+)NC group; ^##^*P* < 0.01 vs. Zic4(−)NC group. **m** Western blot assay and real-time PCR were used to detect the expression of UPF1 after Zic4 overexpression or knockdown. **n** Zic4 bound to the promoter of UPF1 in U87 and U251 cells. Schematic representation of the human UPF1 promoter region 3000 bp upstream of the transcription start site (TSS), which was designated as +1. Putative Zic4 binding site was indicated. PCR was conducted with the resulting precipitated DNA. **o** Real-time PCR was used to detect the expression of Linc-00313 after Zic4 overexpression or knockdown. **p** Zic4 bound to the promoter of Linc-00313 in U87 and U251 cells. Schematic representation of the human Linc-00313 promoter region 3000 bp upstream of the transcription start site (TSS), which was designated as +1. Putative Zic4 binding site was indicated. PCR was conducted with the resulting precipitated DNA. **q** Western blot assay and real-time PCR were used to detect the expression of SHCBP1 after Zic4 overexpression or knockdown. **r** Zic4 bound to the promoter of SHCBP1 in U87 and U251 cells. Schematic representation of the human SHCBP1 promoter region 3000 bp upstream of the transcription start site (TSS), which was designated as +1. Putative Zic4 binding site was indicated. PCR was conducted with the resulting precipitated DNA

The expression of Zic4 in glioma tissues and glioma cells was significantly increased compared with that in normal brain tissues and HA cells. Furthermore, the TCGA database was used to predict the expression of Zic4. Results showed that Zic4 was upregulated in glioma (Fig. 5a–c).

The mRNA and protein expression of Zic4 in the Agomir-342-3p group (Fig. 5d, g) and Agomir-485-5p group (Fig. 5e, h) were significantly lower than that in the Agomir-342-3p-NC group (Fig. 5d, g) and Agomir-485-5p-NC group (Fig. 5e, h). The Antagomir-342-3p group (Fig. 5d, g) and Antagomir-485-5p group (Fig. 5e, h) had the opposite effect. The miRanda database was used to predict the binding sites of miR-342-3p and miR-485-5p in the 3′UTR of Zic4 (Fig. S1C). As shown in Fig. 5f, i, compared with the Zic4-Wt + Agomir-342-3p-NC group (Fig. 5f) and Zic4-Wt + Agomir-485-5p-NC group (Fig. 5i), the luciferase activity was significantly decreased in the Zic4-Wt + Agomir-342-3p group (Fig. 5f) and Zic4-Wt + Agomir-485-5p group (Fig. 5i).

As shown in Fig. 5j–l, the cell proliferation, migration, and invasion of Zic4(+) group were significantly increased, cell apoptosis were significantly decreased compared with the Zic4(+)NC group. And the Zic4(−) group had the opposite effect.

JASPAR CORE database was used to predict the binding sites of Zic4 in the promotor region of Linc-00313 and UPF1 (Fig. S1D). And the expression of Linc-00313 and UPF1 were significantly increased in the Zic4(+) group compared with the Zic4(+)NC group. The Zic4(−) group had the opposite effect. Furthermore, the results of ChIP experiment showed that Zic4 had binding sites in the promoter region of Linc-00313 and UPF1 (Fig. 5m–p).

JASPAR CORE database was used to predict the binding sites of Zic4 in the promotor region of SHCBP1 (Fig. S1E). The expression of SHCBP1 were significantly increased in the Zic4(+) group compared with the Zic4(+)NC group. And the Zic4(−) group had the opposite effect. The results of ChIP experiment showed that Zic4 had binding sites in the promoter region of SHCBP1 (Fig. 5q, r).

### MiR-342-3p and miR-485-5p both played important roles in the regulation of the target gene Zic4 and the biological behaviors of glioma cells

**Fig. 6:**
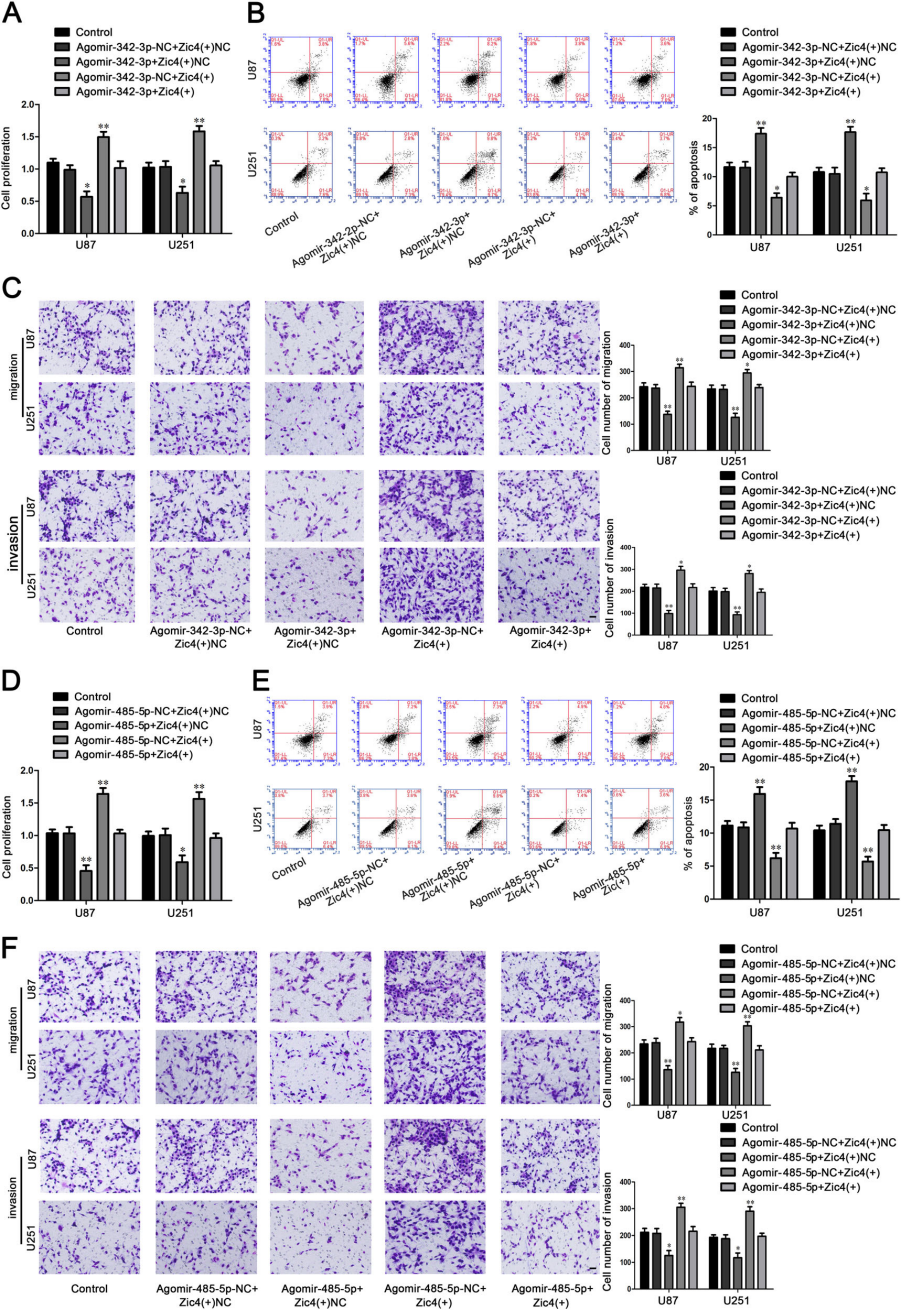
Zic4 mediated tumor-suppressive effects of miR-342-3p and miR-485-5p. **a** CCK8 assay to evaluate the effect of miR-342-3p and Zic4 on cell proliferation of U87 and U251 cells. **b** Flow cytometry analysis to evaluate the effect of miR-342-3p and Zic4 on cell apoptosis of U87 and U251 cells. **c** Transwell assay to evaluate the effect of miR-342-3p and Zic4 on the cell migration and invasion of U87 and U251 cells. Data are presented as the mean ± SD (*n* = 3, each group). Scale bars represent 40 μm. *P < 0.05 vs. Agomir-342-3p + Zic4(+)NC group, ^##^*P* < 0.01 vs. Agomir-342-3p-NC + Zic4(+) group. **d** CCK8 assay to evaluate the effect of miR-485-5p and Zic4 on cell proliferation of U87 and U251 cells. **e** Flow cytometry analysis to evaluate the effect of miR-485-5p and Zic4 on cell apoptosis of U87 and U251 cells. **f** Transwell assay to evaluate the effect of miR-485-5p and Zic4 on the cell migration and invasion of U87 and U251 cells. Data are presented as the mean ± SD (*n* = 3, each group). Scale bars represent 40 μm. *P < 0.05 vs. Agomir-485-5p + Zic4(+)NC group, ^##^*P* < 0.01 vs. Agomir-485-5p-NC + Zic4(+)group

U87 and U251 cells were cotransfected with miR-342-3p or miR-485-5p and Zic4. As shown in Fig. 6a–c, the cell proliferation, migration, and invasion in Agomir-342-3p + Zic4(+)NC group were significantly decreased, the apoptosis were significantly increased compared with the Agomir-342-3p-NC + Zic4(+)NC group. And the Agomir-342-3p-NC + Zic4(+) group had the opposite effect. The cell proliferation, migration and invasion in Agomir-485-5p + Zic4(+)NC group were significantly decreased, the apoptosis were significantly increased compared with the Agomir-485-5p-NC + Zic4(+)NC group. And the Agomir-485-5p-NC + Zic4(+) group had the opposite effect (Fig. 6d–f).

### The endogenous expression of SHCBP1 in glioma tissues and cells, and its regulation of the biological behavior of glioma cells

**Fig. 7:**
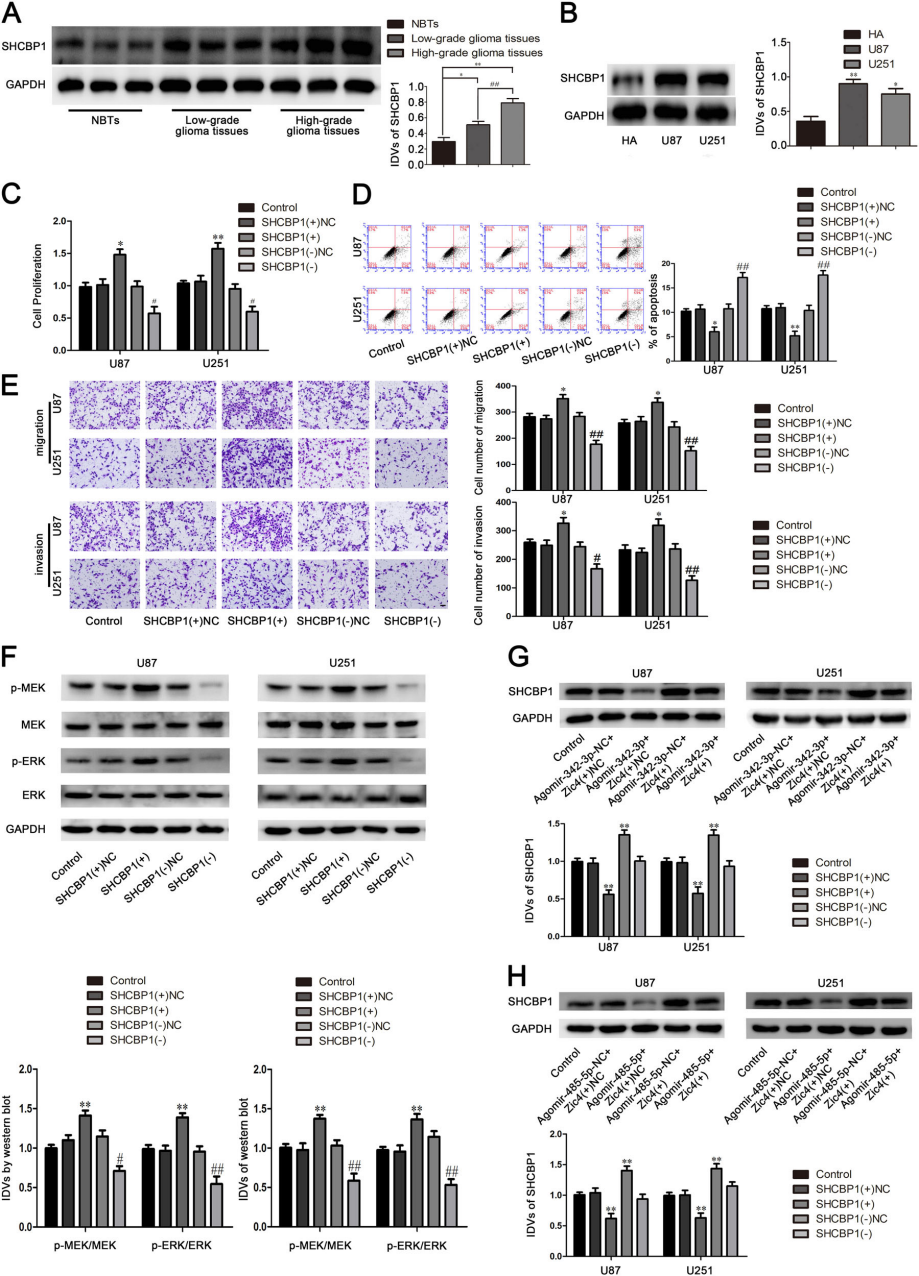
SHCBP1 endogenous expression and effect on proliferation, migration, invasion, and apoptosis of U87 and U251. **a** SHCBP1 protein expression levels in normal brain tissues (NBTs), and glioma tissues of different grades. Data are presented as the mean ± SD (*n* = 9, each group). **P < 0.01 vs. NBTs group; ^#^*P* < 0.05 vs. low-grade glioma tissues group. **b** The expression of SHCBP1 in human astrocytes (HA) and glioblastoma cell lines (U87 and U251). **c** CCK-8 assay was used to measure the effect of SHCBP1 on the proliferation of U87 and U251 cells. **d** The apoptotic percentages of U87 and U251 were detected after SHCBP1 overexpression or knockdown. **e** Transwell assays were used to measure the effect of SHCBP1 on cell migration and invasion of U87 and U251 cells. Data are presented as the mean ± SD (*n* = 3, each group). **P < 0.01 vs. SHCBP1(+)NC, ^#^*P* < 0.05 vs. SHCBP1(−)NC. **f** Western blot assay of the p-MEK-3/MEK-3 and p-ERK-1/ERK-1 expression regulated by SHCBP1. **g** Western blot assay were used to detect the SHCBP1 expression regulated by miR-342-3p and Zic4. **h** Western blot assay were used to detect the SHCBP1 expression regulated by miR-485-5p and Zic4. Data are presented as the mean ± SD. **P < 0.01 vs. Agomir-485-5p + Zic4(+)NC group, ^##^*P* < 0.01 vs. Agomir-485-5p-NC + Zic4(+) group

In this study we found that the expression of SHCBP1 in glioma tissues and glioma cells were significantly increased compared with that in normal brain tissues and HA cells (Fig. 7a, b).

As shown in Fig. 7c-e, after SHCBP1 overexpression or knockdown, the cell proliferation, migration, and invasion in SHCBP1(+) group were significantly increased, the apoptosis were significantly decreased compared with the SHCBP1(+)NC group. And the SHCBP1(−) group had the opposite effect.

As shown in Fig. 7f, overexpression of SHCBP1 activated the MEK/ERK signaling pathway, and knockdown of SHCBP1 inhibited the MEK/ERK signaling pathway. The expression of p-MEK-3/MEK-3 and p-ERK-1/2/ERK-1/2 in the SHCBP1(+) group were significantly increased compared with the SHCBP1(+)NC group. And the SHCBP1(−) group had the opposite effect.

To detect whether miR-342-3p and miR-485-5p reduced SHCBP1 expression by downregulating Zic4 expression, we cotransfected the U87 and U251 cells with miR-342-3p or miR-485-5p and Zic4. As shown in Fig. 7g, h, the expression of SHCBP1 in the Agomir-342-3p + Zic4(+)NC group (Fig. 7g) and Agomir-485-5p + Zic4(+)NC group (Fig. 7h) were significantly decreased compared with the Agomir-342-3p-NC + Zic4(+)NC group (Fig. 7g) and Agomir-485-5p-NC + Zic4(+)NC group (Fig. 7h). And the Agomir-342-3p-NC + Zic4(+) group (Fig. 7g) and Agomir-485-5p-NC + Zic4(+) group (Fig. 7h) had the opposite effect.

### Knockdown of Linc-00313 combined with overexpression of miR-342-3p and miR-485-5p suppressed tumor growth and induced the longest survival time in nude mice

**Fig. 8:**
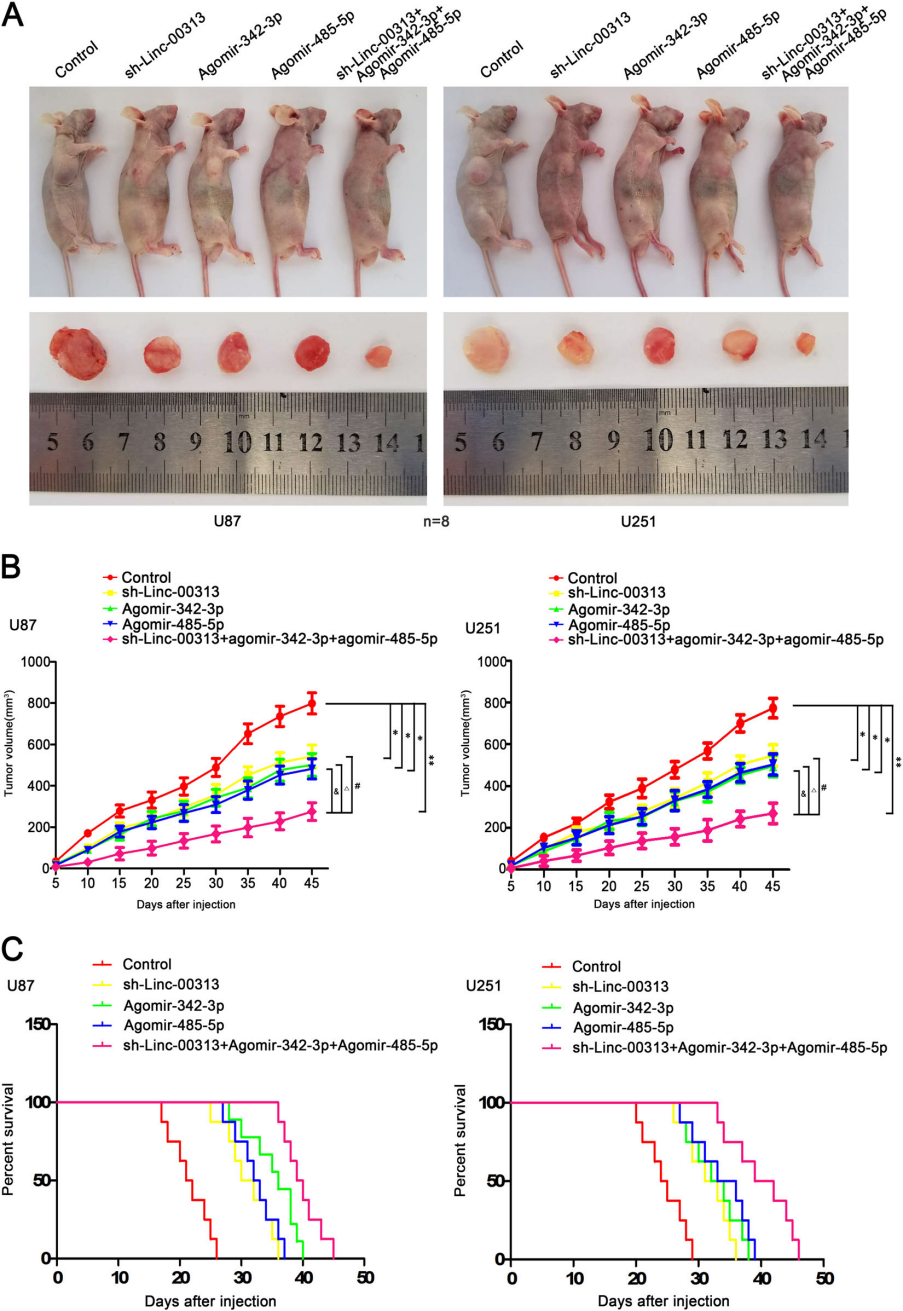
Tumor xenograft studies. **a** The nude mice carrying tumors from respective groups were shown. The sample tumors from respective group were shown. **b** Tumor growth curves were shown. Tumor volume was calculated every 5 days after injection, and the tumor was taken after 45 days. **c** Survival curves from representive nude mice injected into the right striatum were shown (*n* = 8, each group)

Tumor xenograft model in nude mice was established to determine the functions of Linc-00313, miR-342-3p, and miR-485-5p. As shown in Fig. 8a, b, the tumor volume in sh-Linc-00313 group, Agomir-342-3p group, Agomir-485-5p group and sh-Linc-00313 + agomir-342-3p + agomir-485-5p group were smaller than those in control group (*n* = 8). And the tumor volume in sh-Linc-00313 + agomir-342-3p + agomir-485-5p group were the smallest. The nude mice in the sh-Linc-00313 + agomir-342-3p + agomir-485-5p group had the longest survival time (Fig. 8c).

## Discussion

This study first showed that UPF1 and Linc-00313 were highly expressed in glioma tissues and U87 and U251 cell lines. The respective knockdown of UPF1 and Linc-00313 significantly inhibited cell proliferation, migration, and invasion, as well as promoted apoptosis in U87 and U251. Studies have shown that the RNA-binding protein UPF1 plays an important role in cell-cycle progression and DNA damage^26^. Furthermore, Oncomine database revealed that UPF1 was upregulated in glioblastoma tissues which corroborated the findings of this study.

LncRNA is involved in many important regulatory processes such as chromosome silencing, genomic imprinting, transcriptional activation^27,28^. LncRNA also plays a significant role in the tumorigenesis and development of brain gliomas or acts as a biomarker^29–34^. Similar to our findings, Linc-00313 had been shown to be upregulated in lung cancer and is a diagnostic biomarker for early stage of lung cancer. The high expression of Linc-00313 is associated with poor prognosis in patients with lung cancer^15,16^. RNA-binding proteins can affect the biological functions of cells by regulating the stability of lncRNAs. For example, all three RNA-binding proteins Nanog, Sox2, and Fgf4 can bind to lncRNA-TUNA and promote neural differentiation of mouse embryonic stem cells^35^. In the present study, the binding site of UPF1 and Linc-00313 was predicted with the Starbase database. Based on the prediction, RIP and half-life assays were performed to confirmed that UPF1 could bind to Linc-00313. Furthermore, the binding enhanced the stability and increased the expression of Linc-00313, regulated the biological behaviors of glioma cells.

Our study showed that miR-342-3p and miR-485-5p were downregulated in glioma tissues and U87 and U251 cells. The respective overexpression of miR-342-3p and miR-485-5p could inhibit the cell proliferation, migration, and invasion of U87 and U251 cells, as well as promote their apoptosis. The silencing of miR-342-3p and miR-485-5p had the opposite effect. MiR-342-2p is located in the intron of its host gene *EVL*, and its expression is the same as the mRNA expression of the *EVL* gene^36^. Consistent with the results of this study, miR-342-3p was downregulated in brain gliomas^18^ and could inhibit the proliferation, migration, and invasion of glioma cells^19^. In addition, the expression of miR-342-3p was also found to be downregulated in colorectal cancer, breast cancer, and gallbladder cancer^17,37–39^. Other studies had shown that miR-342-3p can influence the sensitivity of anticancer chemotherapy agents by regulating histone methylation^40,41^. MiR-485-5p is downregulated in glioma tissues and cell lines, and the overexpression of miR-485-5p could inhibit the proliferation, migration, and invasion of glioma cells^20^. In addition, miR-485-5p was also downregulated in gastric cancer^42^ and breast cancer^43^. MiR-485-5p could also significantly inhibit the cell invasion and proliferation ability of melanoma^44^.

The Starbase database was used to predict the binding sites of miR-342-3p, miR-485-5p with Linc-00313. Based on the predictions, reporter vectors construction and luciferase assays were performed to confirm that Linc-00313 could bind to miR-342-3p and miR-485-5p, respectively. Knockdown of Linc-00313 significantly upregulated the expression of miR-342-3p and miR-485-5p, which led to inhibition of the cell proliferation, migration, and invasion of glioma cells, as well as promotion of cell apoptosis. The study further demonstrated that Zic4 was highly expressed in glioma tissues and U87 and U251 cells The silencing of Zic4 expression could inhibit the cell proliferation, migration, and invasion of U87 and U251 cells, as well as promote cell apoptosis. The overexpression of Zic4 had the opposite effect. The binding sites of miR-342-3p and miR-485-5p were predicted to located in the 3′UTR of Zic4 with miRanda database. Reporter vectors construction and luciferase assays were performed to confirm the binding sites between miR-342-3p or miR-485-5p and Zic4, respectively. The simultaneous overexpression of Zic4 and miR-342-3p or miR-485-5p could mediate the biological effects on glioma cells caused by the overexpression of miR-342-3p, miR-485-5p, or Zic4 alone. These results indicated that the effects of miR-342-3p or miR-485-5p overexpression on the biological behaviors of glioma cells were due to the enhanced negative regulation of their downstream target gene Zic4. The knockdown of Linc-00313 significantly upregulated the expression of miR-342-3p and miR-485-5p, which led to the inhibition of the cell proliferation, migration, and invasion of glioma cells, and promote apoptosis of U87 and U251. The knockdown of Linc-00313 combined with the overexpression of miR-342-3p or miR-485-5p significantly inhibited the expression of Zic4, the cell proliferation, migration and invasion of glioma cells, as well as promoted apoptosis. These results indicated that Linc-00313 could impact the negative regulation of miR-342-3p and miR-485-5p on their target gene Zic4 by binding to miR-342-3p and miR-485-5p, and then affect the biological behaviors of glioma cells.

LncRNA can bind to miRNA and act as its “molecular sponge”. LncRNA can also function as competing endogenous RNA (ceRNA), which affects the regulation of miRNA on downstream target genes. LncRNA has become one of the “bridges” for the regulation of miRNAs and downstream target genes. The long non-coding RNA-H19 could act as a ceRNA, bind to miR-106a-5p and upregulates E2F3, thereby promoting glucose metabolism and cell growth in malignant melanoma cells^45^. The present study found that the MRE sequence (GUGUGAG, CAGCCUC) in which miR-342-3p and miR-485-5p bind to Linc-00313 is the same as that bind to the 3’UTR of Zic4, respectively. These results indicated that Linc-00313 could function as a ceRNA and bind to miR-342-3p and miR-485-5p, respectively, attenuating the negative regulation of miR-342-3p and miR-485-5p on the downstream target gene Zic4, and then affected the biological behaviors of glioma cells.

The oncogene SS18-SSX1 can promote the tumorigenesis and development of synovial sarcoma by enhancing the expression of SHCBP1^46,47^. The present study demonstrated that SHCBP1 was upregulated in glioma tissues and in U87 and U251 cells. The overexpression of SHCBP1 could promote the cell proliferation, migration, and invasion of U87 and U251 cells, and inhibit cell apoptosis. Silence of SHCBP1 had the opposite effect. The JASPAR CORE database was used to predict the Zic4 binding sites in the promoter region of SHCBP1. ChIP experiments were performed to demonstrate that Zic4 could bind to the SHCBP1 promoter region and regulate its transcription. It showed that Zic4 could regulate SHCBP1 at the transcriptional level. The simultaneous overexpression of Zic4 and miR-342-3p or miR-485-5p could mediate the effects on SHCBP1 expression caused by the overexpression of miR-342-3p, miR-485-5p, or Zic4 alone. These results suggested that the overexpression of miR-342-3p or miR-485-5p enhanced the negatively regulation of Zic4, which could reduce the transcription and expression of the Zic4 downstream target gene SHCBP1. And then led to the inhibition of the malignant biological behaviors of glioma cells.

The MEK/ERK signaling pathway can transmit extracellular signals to the nucleus via cell membrane receptors and the its activation can promote the proliferation of many cancer cell types and enhance other biological functions. For example, HER2 can promote the proliferation and invasion of non-small cell lung cancer by activating the MEK/ERK signaling pathway^48^. The inhibition of CCN1 in leukemia cells can inhibit cell growth by inhibiting the activation of the MEK/ERK signaling pathway^49^. The present study demonstrated that the overexpression of SHCBP1 increased the expression of p-MEK/MEK and p-ERK/ERK, and activated the MEK/ERK signaling pathway, which promoted the proliferation, migration, and invasion of glioma cells, and inhibited their apoptosis^50,51^. Knockdown of SHCBP1 had the opposite effect. These results showed that SHCBP1 could regulate the biological behaviors of glioma cells itself, as well as through the MEK/ERK signaling pathway.

Interestingly, the binding sites of Zic4 in the promoter regions of UPF1 and Linc-00313 were predicted via the JASPAR CORE database and confirmed with ChIP experiments. The overexpression of Zic4 significantly increased the expression of UPF1 and Linc-00313, whereas silence of Zic4 expression had the opposite effect. In addition, all three molecules were highly expressed in glioma tissues and U87 and U251 cells. These results suggested that UPF1/Linc-00313/miR-342-3p(miR-485-5p)/Zic4 formed a positive-feedback loop to regulate the biological behaviors of glioma cells.

Finally, a nude mouse xenograft model was used in this study to demonstrate that sh-Linc-00313, Agomir-342-3p, or Agomir-485-5p alone, as well as the combination of sh-Linc-00313, Agomir-342-3p and Agomir-485-5p, could significantly reduced the tumor volumes in nude mice with the xenografts of U87 and U251 cells. The survival of nude mice was also extended, and the combination of sh-Linc-00313 + Agomir-342-3p + Agomir-485-5p resulted in the smallest tumor volumes and longest survival in the nude mice. These results suggested that the combination of sh-Linc-00313, Agomir-342-3p, and Agomir-485-5p could have potential clinical application values.

In summary, this study showed that UPF1 could bind to Linc-00313 and enhance its stability. Linc-00313 inhibited the negative regulation of miR-342-3p and miR-485-5p on their common downstream target gene Zic4, which further regulated the transcription and expression levels of SHCBP1. In addition, SHCBP1 could regulate the biological behaviors of glioma cells by activating the MEK/ERK signaling pathway. At the same time, Zic4 could positively regulate the transcriptional expression of UPF1 and Linc-00313, thus formed a positive-feedback loop that regulated the biological behaviors of glioma cells. The results could provide new theoretical and experimental evidence for the study of the mechanism of glioma tumorigenesis and development, as well as new targets for the treatment of glioma.

## Methods and materials

### Human glioma tissue samples and cell culture

Human glioma and normal brain tissues were collected form patients undergoing surgery at the Department of Neurosurgery, Shengjing Hospital of China Medical of University. NBTs were the rejected material from surgeries of brain trauma or epilepsy. And the approval from the Hospital Ethical Committee was obtained. Human astrocyte (HA) cells were purchased from Scien-Cell Research Laboratories and cultured in RPMI-1640 culture medium with 10% fetal bovine serum. And the Human glioblastoma (GBM) cell lines U87, U251, and human embryonic kidney (HEK) 293T cells were purchased from the Shanghai Institutes for Biological Sciences Cell Resource Center and cultured in Dulbecco’s modified Eagle medium of high glucose supplemented with 10% fetal bovine serum. The cells were all maintained in a humidified incubator at 37 °C with 5% CO_2_.

### RNA extraction and quantitative reverse transcription-PCR

Total RNA was isolated from the cells by Trizol reagent. The expression of UPF1, Linc-00313, and SHCBP1 was detected by One-Step SYBR PrimeScript RT-PCR Kit with 7500 Fast RT-PCR System. And the reverse transcription of miR-342-2p/miR-485-5p were achieved by TaqMan MicroRNA Reverse Transcription kit. The expression of miR-342-2p and miR-485-5p were detected with TaqMan Universal Master Mix II. Primers and probes used in this study are shown in Table S1.

### Cell transfection

Short-hairpin RNA against UPF1, Linc-00313, Zic4, or SHCBP1 gene, as well as their nontargeting sequences were reconstructed in pGPU6/GFP/Neo vector. The full-length UPF1, Zic4, or SHCBP1 gene were constructed in pIRES2-EGFP. And the empty vectors were used as NCs. Cells were seeded in a 24-well plate. We used Lipofectamine 3000 reagent and Opti-MEM I to transfect cells with the plasmids according to the manufacturer’s instructions. G418 was used to select the stable transfected cells. For the transient transfection assays, Agomir-342-3p, Antagomir-342-3p, Agomir-485-5p, Antagomir-485-5p, and and their NC sequence were synthesized. Cells were collected 48 h after transfection. Sequences of the small hairpin RNA template are shown in Table S2.

### Western blot analysis

Total proteins were extracted from the HA, U87 and U251 cells. Then we subjected the proteins to sodium dodecyl sulphate polyacrylamide gel electrophoresis and electrophoretically transferred to polyvinylidene fluoride membranes. Membranes were incubated in 5% nonfat milk dissolved in Tween-Tris-buffered saline for 2 h at room temperature and then incubated with primary antibodies overnight at 4 °C. Then incubated by appropriate correlated horseradish peroxidase-conjugated secondary antibody at room temperature for 2 h. Finally, scanned by ChemImager 5500 V2.03 software after visualized by enhanced chemiluminescence.

### Cell viability analysis

Cells were seeded in 96-well plates. Then 20 μL of CCK-8 were added into per well after 48 h and incubated at 37 °C for 2 h. The absorbance was measured at 450 nm with a spectrophotometer.

### Quantization of apoptosis by flow cytometry

After washing with PBS twice, we stained the cells with Annexin V-FITC/PI according to the manufacturer’s instructions. Then cells were analyzed by flow cytometry and apoptotic fractions were acquired.

### Cell migration and invasion assays

Transwell chambers with a pore size of 8 µm were used in cell migration and invasion assays. Cells were resuspended in 100 μL serum-free medium and were seeded into the upper chamber. The lower chamber was filled with 600 µL GBM cell-conditioned medium. After 48 h at 37 °C, we fixed and stained the cells. Five randomly fields were counted and photos were taken under a microscope.

### Reporter vectors construction and luciferase assays

HEK-293T cells were seeded in a 96-well plate. Linc-00313 sequence, Zic4-3′UTR sequence were amplified by PCR and cloned into pmirGLO Dual-luciferase miRNA Target Expression Vectors as well as their mutant sequences of miR-342-3p/miR-485-5p binding sites. Then we, respectively, cotransfected the HEK-293T cells with wild-type pmirGLO-Linc-00313, mutant-type pmirGLO-Linc-00313 (or Zic4-3′UTR-Wt, Zic4-3′UTR-Mut) reporter plasmid and agomir-342-3p/agomir-485-5p. After 48 h, with normalizing to renilla luciferase activity. The luciferase activities can be calculated after performed with the Dual-Lucifer Reporter Assay System according to the manufacturer’s instructions.

### Human lncRNA microarrays

LncRNA analysis, sample preparation and microarray hybridization were performed by Kangchen Bio-tech.

### RNA immunoprecipitation (RIP)

Whole-cell lysate was incubated with RIP buffer containing magnetic beads conjugated with human anti-UPF1 antibody. Normal mouse IgG was used as a NC. Samples were incubated with Proteinase K buffer and then immunoprecipitated RNA was isolated and analyzed by real-time PCR to demonstrate the presence of binding targets.

### Half-life assay

Cells were seeded into a six-well plate. Each well was added with 5 μg/ml Actinomycin D. Then cells were collected at 0, 2, 4, 6, 8, and 10 h after the addition of Actinomycin D. And real-time PCR was used to measure the stability of Linc-00313.

### Chromatin immunoprecipitation (ChIP) assay

The Simple Chip Enzymatic Chromatin IP kit was used for ChIP assay according to the manufacturer’s instructions. U87/U251 cells were crosslinked with 1% formaldehyde for 10 min and added glycine for 5 min at room temperature to quenched cross-link. Then cells were collected in lysis buffer with 1% phenylmethanesulfonyl fluoride. Lysates (2%) was used as an input reference. And the other lysates was incubated with 3 μg of anti-Zic4 antibody or normal rabbit IgG with rotation. DNA cross-links were reversed by 5 mol/l NaCl and proteinase K at 65 °C for 2 h. DNA was amplified by PCR with their specific primers. And the primers used for ChIP PCR are shown in Table S3.

### Tumor xenograft implantation

Four-week-old male BALB/C nude mice were purchased from Cancer Institute of the Chinese Academy of Medical Science. Then we divided the mice into five groups (*n* = 8). Each group of mice were injected different kind of suspending cells (3 × 10^5^) subcutaneously into the right flank. Tumor nodules were estimated every 5 days with caliper. And the number of survived nude mice was registered and survival analysis was performed with Kaplan–Meier survival curve. After 45 days, the mice were sacrificed. Then we isolated the tumors and took photos. All animal experiments were complied with the guidelines of the Animal Welfare Act and were reviewed and approved by the Administrate Panel on Laboratory Animal Care of China Medical University.

### Statistical analysis

Experimental data were presented as means ± standard deviation. Differences were analyzed by SPSS 18.0 statistical software with the Student’s *t* test or one-way ANOVA. Differences were considered significant if *P* < 0.05.

## Supplementary information

supplemental Figure Legends

Table S1-3

Figure-S1

Figure-S2

Figure-S3

Figure-S4

Figure-S5

Figure-S6

Figure-S7

Figure-S8

Figure-S9

Figure-S10

Figure-S11

Figure-S12

Figure-S13

## Data Availability

The data supporting the conclusion of this research has been included in this published article and its additional files.
