# Supplementary material for: UPF1 regulates the malignant biological behaviors of glioblastoma cells via enhancing the stability of Linc-00313
Source: Cell Death Dis. 2019 Aug 19;10(9):629. doi: 10.1038/s41419-019-1845-1 (PMC6700115; doi:10.1038/s41419-019-1845-1)
Supplement: Supplementary file 1 — supplemental Figure Legends [file 41419_2019_1845_MOESM1_ESM.docx]

**Figure-SI Legends**

**Fig-S1 The binding sites predicted by databases. (A)**The binding sites of UPF1 and Linc00313 were predicted by catRAPID and RPISeq databases. **(B)**The binding sites of Linc00313 and miR-342-3p, miR-485-5p were predicted by starbase database. **(C)**The binding sites of miR-342-3p, miR-485-5p and Zic4 were predicted by miRanda database. **(D)**The binding sites of Zic4 and Linc-00313, UPF1 were predicted by JASPAR CORE database. **(E)**The binding sites of Zic4 and SHCBP1 were predicted by JASPAR CORE database.

**Fig-S2 The Zic4 expression regulated by UPF1, Linc-00313, miR-342-3p and miR-485-5p.** **(A)**Real-time PCR and **(B)**Western blot assay were used to detect the Zic4 expression after UPF1 knockdown. **(C)**Real-time PCR and **(D)**Western blot assay were used to detect the Zic4 expression after Linc-00313 knockdown. **(E)**Real-time PCR and **(F)**Western blot assay were used to detect the Zic4 expression regulated by Linc-00313 and miR-342-3p. **(G)**Real-time PCR and **(H)**Western blot assay were used to detect the Zic4 expression regulated by Linc-00313 and miR-485-5p. ***P*< 0.01, ^#^*P*< 0.05.

**Fig-S3 Downregulation of UPF1 Inhibited the Expression Level of Linc-00313. (A)**LncRNA gene expression profiles as obtained from samples in two groups as indicated. Red indicates high relative expression and green indicates low relative expression.

**Fig-S4 The transfection efficiency of UPF1, Linc-00313, miR-342-3p, miR-485-5p, Zic4 and SHCBP1. (A)(B)(C)(D)**The transfection efficiency of UPF1 in U87 and U251 cells. **(E)(F)**The transfection efficiency of Linc-00313 in U87 and U251 cells. **(G)(H)**The transfection efficiency of miR-342-3p in U87 and U251 cells. **(I)(J)**The transfection efficiency of miR-485-5p in U87 and U251 cells. **(K)(L)(M)(N)**The transfection efficiency of Zic4 in U87 and U251 cells. **(O)(P)(Q)(R)**The transfection efficiency of SHCBP1 in U87 and U251 cells. ***P*< 0.01, ^#^*P*< 0.05.

**Fig-S5 The expression of Zic4 and SHCBP1 in the nude mice tumor. (A)**The expression of Zic4 in the nude mice tumor. **(B)**The expression of SHCBP1 in the nude mice tumor.

**Fig-S6 The expression and survival curve predicted by databases. (A)**The survival curve of UPF1 predicted by GEPIA databases. **(B)**The survival curve of Linc-00313 predicted by GEPIA databases. **(C)**The survival curve of Zic4 predicted by GEPIA databases. **(D)**The survival curve of SHCBP1 predicted by GEPIA databases. **(E)**The expression of UPF1 predicted by Oncomine databases.. **(F)**The expression of Zic4 predicted by Oncomine databases. **(G)**The expression of SHCBP1 predicted by Oncomine databases.

**Fig-S7 The schematic diagram of the oncogenic role of UPF1, Linc-00313, miR-342-3p, miR-485-5p, Zic4 and SHCBP1 in Glioma cells.**

**Fig-S8 The result of apoptotic percentages from Fig.1-Fig.8.**

**Fig-S9 The endogenous expression of UPF1 detected by Immunohistochemical.**

**Fig-S10 Downregulation of Linc-00313 promoted the Expression Level of miR-342-3p and miR-485-5p. (A)**miRNA gene expression profiles as obtained from samples in two groups as indicated. Red indicates high relative expression and green indicates low relative expression.

**Fig-S11 The expression of UPF1 was measured by Western blot after Linc-00313 was RIPed.**

**Fig-S12 The stability of Linc-00313 after UPF1-Mut. (A)**Linc-00313 RNA half-life measured by qRT-PCR after actinomycin D treatment in U87 cells. **(B)**Linc-00313 RNA half-life measured by qRT-PCR after actinomycin D treatment in U251 cells.

**Fig-S13 The correlation of UPF1 and Linc00313 as well as Zic4 and SHCBP1 predicted by databases. (A)**The correlation of UPF1 and Linc00313 predicted by GEPIA databases. **(B)**The correlation of Zic4 and SHCBP1 predicted by GEPIA databases.
