# Supplementary material for: UPF1 regulates the malignant biological behaviors of glioblastoma cells via enhancing the stability of Linc-00313
Source: Cell Death Dis. 2019 Aug 19;10(9):629. doi: 10.1038/s41419-019-1845-1 (PMC6700115; doi:10.1038/s41419-019-1845-1)
Supplement: Supplementary file 2 — Table S1-3 [file 41419_2019_1845_MOESM2_ESM.docx]

| Primer or Probe | Gene | Sequence (5'->3') or Assay ID |
| --- | --- | --- |
| Primer | UPF1 | F: GTCTTCCTCCTCGGCTTCAT |
|  |  | R: GCTGTCCCAGTTGATGTCCT |
|  | Linc-00313 | F: CTAGAGAGCTGGCGCACCTCC |
|  | Zic4  SHCBP1 | R: GCGGCCGCTCCTCATG  F:CCCTCTGGGAAAAGGCTCAA  R:CATGGTGTCCAGAGCTGCTA  F:TGAGCGATTCAGAGCCTATCA  R:ATTAGTGTGCCAGACTGCCC |
|  | GAPDH | F: GGTGAAGGTCGGAGTCAACG |
|  |  | R: CCATGTAGTTGAGGTCAATGAAG |
|  | U6 | F:CTCGCTTCGGCAGCACA  R: AACGCTTCACGAATTTGCGT |
| Probe | MiR-342-3p  MiR-485-5p | 002260(Applied biosystems)  001036(Applied biosystems) |
|  | U6 | 001973(Applied biosystems) |

Table 1. Primers and probes used for RT-qPCR.

One-Step SYBR PrimeScript RT-PCR cycling conditions were as follows: 5 minutes at 42°C, 10 seconds at 95°C, 40 cycles of 3 seconds at 95°C, and 30 seconds at 60°C.

The reverse transcription was set as follows: 30 minutes at 16°C, 30 minutes at 42°C, and 5 minutes at 85°C. PCR conditions were set as follows: 2 minutes at 50°C, 10 minutes at 95°C, 40 cycles of 15 seconds at 95°C and 1 minutes at 60°C.

Table 2. Sequences of shRNA template

| Gene |  | Sequence(5'->3') |
| --- | --- | --- |
| UPF1 | Sence | CACCGCGAGAAGGACTTCATCATCCTTCAAGAGACGATAACTCCTGAAATCCAGCTTTTTTG |
|  | Antisence | GATCCAAAAAAGCTGGATTTCAGGAGTTATCGTCTCTTGAAGGATGATGAAGTCCTTCTCGC |
| Linc-00313 | Sence | CACCGCTTCCTGGATTGCATAAAGG TTCAAGAGAATATCAGCACCAGTGCAGTCCTTTTTTG |
| Zic4  SHCBP1 | Antisence  Sence  Antisence  Sence  Antisence | GATCCAAAAAAGGACTGCACTGGTGCTGATATTCTCTTGAACCTTTATGCAATCCAGGAAGC  CACCGCATTCGCACGTGCACACTAGTTCAAGAGACTAGTGTGCACGTGCGAATGCTTTTTTG  GATCCAAAAAAGCATTCGCACGTGCACACTAGTCTCTTGAACTAGTGTGCACGTGCGAATGC  CACCGGACTGCACTGGTGCTGATATTTCAAGAGAATATCAGCACCAGTGCAGTCCTTTTTTG  GATCCAAAAAAGGACTGCACTGGTGCTGATATTCTCTTGAAATATCAGCACCAGTGCAGTCC |
| NC | Sence | CACCGTTCTCCGAACGTGTCACGTCAAGAGATTACGTGACACGTTCGGAGAATTTTTTG |
|  | Antisence | GATCCAAAAAAGTTCTCCGAACGTGTCACGTAATCTCTTGACGTGACACGTTCGGAGAAC |

Table 3. Primers used for ChIP experiments

| Gene | Binding site or Control | Sequence (5'->3') | Product size (bp) | Annealing temperature (°C) |
| --- | --- | --- | --- | --- |
| UPF1 | PCR1 | F: AATAAGGAACCACGGCAAACTG | 150 | 59.5 |
|  |  | R: CTTCGCTTCCTCTCTGGTAAAGT |  |  |
|  | PCR2 | F: AGACGGGGTTTCACCATGTTAG | 125 | 60.0 |
|  | PCR3 | R: ATATAAAAATTAGGCCGAGCGC  F:ATTAAACCGAAACACCGTCGC  R:GAAGACGGGACGCGCAC | 173 | 59.8 |
| Linc-00313 | PCR1  PCR2  PCR3  PCR4 | F: TCCAAACAGTGGGAGGGTTG  R:AGCAGGAACAGGCTCCAAAG  F:CTTGGGGGAGACAGTTGTGG  R:TGCTGCGGTGAAGTCTAAGG  F:GGTCTAGGCCCTCAACCTTC  R:ACACCTGCCCTCAAAACTGG  F:GGTTCAGCCACTGGGAACTC  R:CAGGAGGCTGAGTCACGG | 114  102  98  144 | 60.2  60.1  59.5  59.6 |
|  | PCR5 | F:TCTGCGCAGCCTCCC  R:GAAGCCACACAGATGCAAGG | 177 | 58.6 |
| SHCBP1 | PCR1 | F:GGAATCGAGTCTCCCAGACA | 156 | 59.0 |
|  |  | R: CGCGCTGTTAAAGGAACC |  |  |
|  | PCR2 | F: CCGAGAGACTTTAGCCCAAGG | 132 | 60.2 |
|  |  | R: CCAGGACACCAAGTGGGATG |  |  |
