# Supplementary figures and images for: UPF1 regulates the malignant biological behaviors of glioblastoma cells via enhancing the stability of Linc-00313
Source: Cell Death Dis. 2019 Aug 19;10(9):629. doi: 10.1038/s41419-019-1845-1 (PMC6700115; doi:10.1038/s41419-019-1845-1)

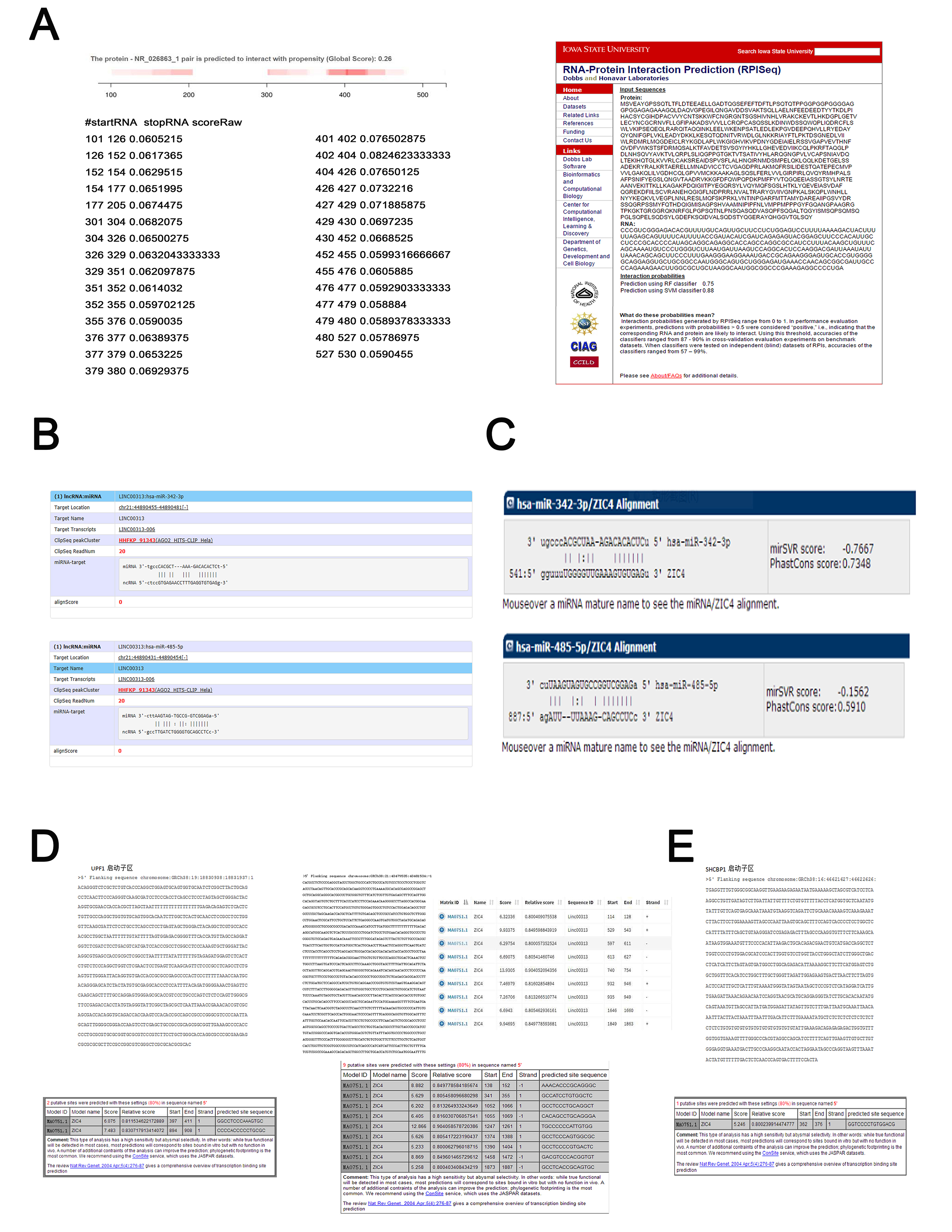

Supplement: Supplementary file 3 — Figure-S1 [file 41419_2019_1845_MOESM3_ESM.tif]

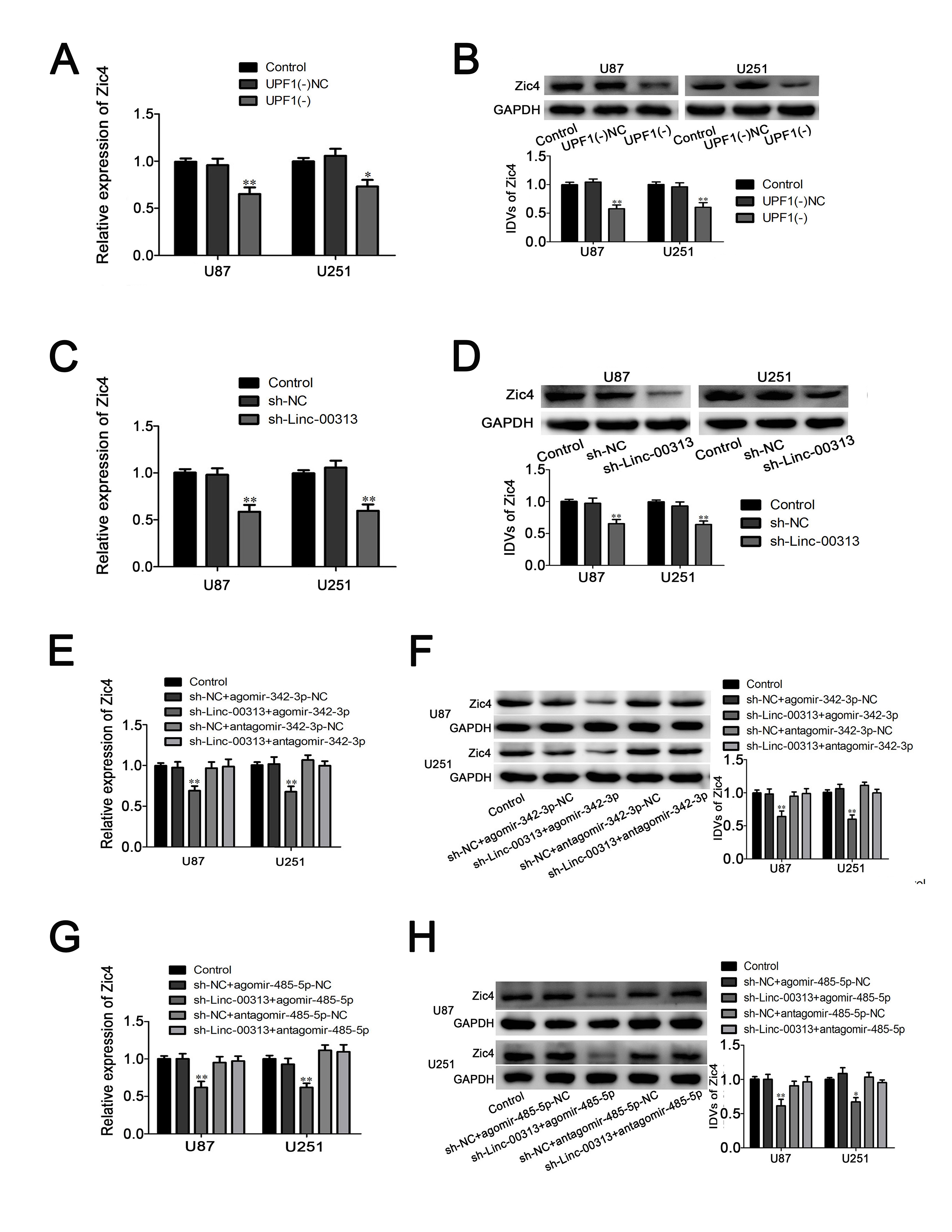

Supplement: Supplementary file 4 — Figure-S2 [file 41419_2019_1845_MOESM4_ESM.tif]

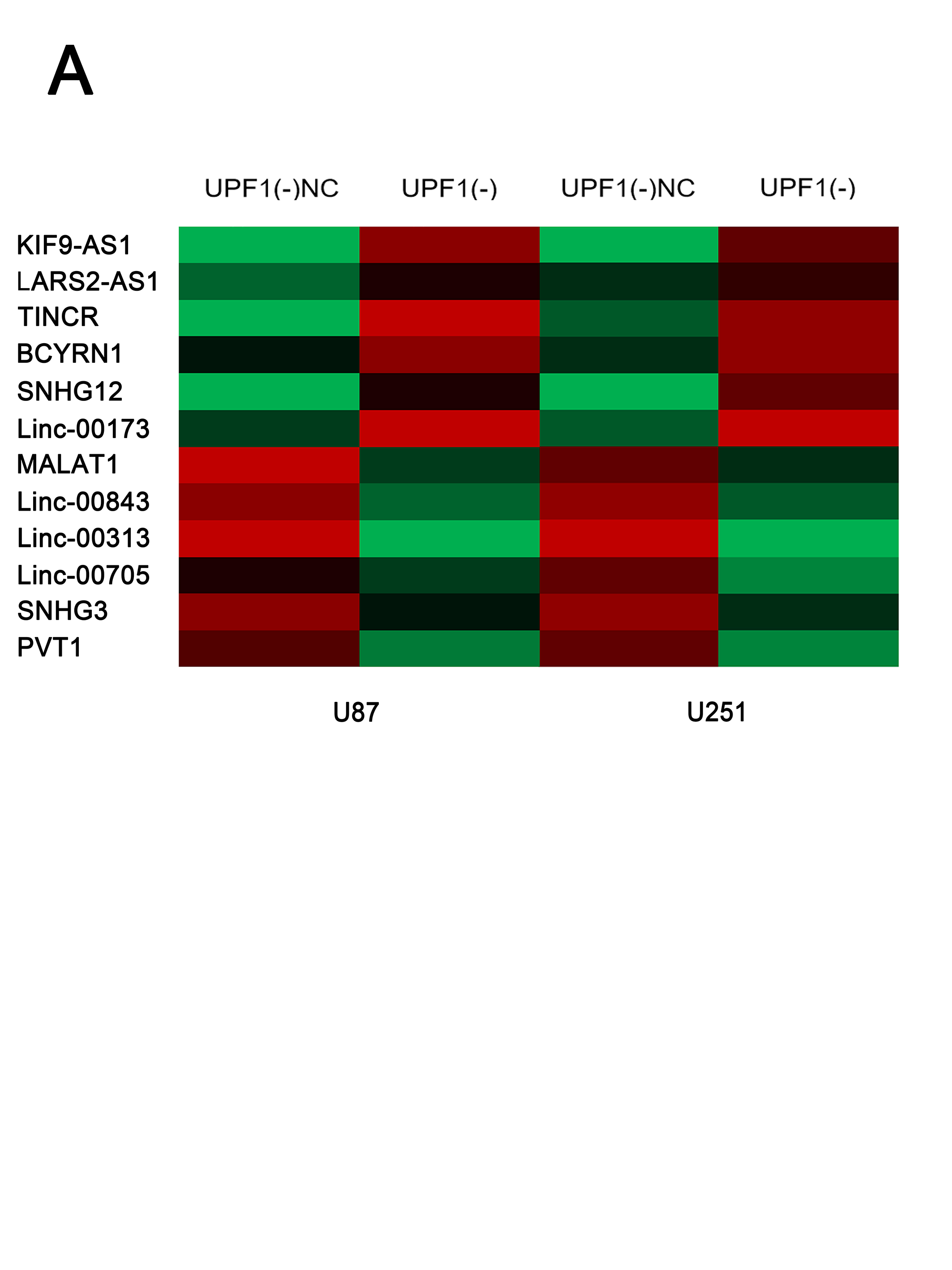

Supplement: Supplementary file 5 — Figure-S3 [file 41419_2019_1845_MOESM5_ESM.tif]

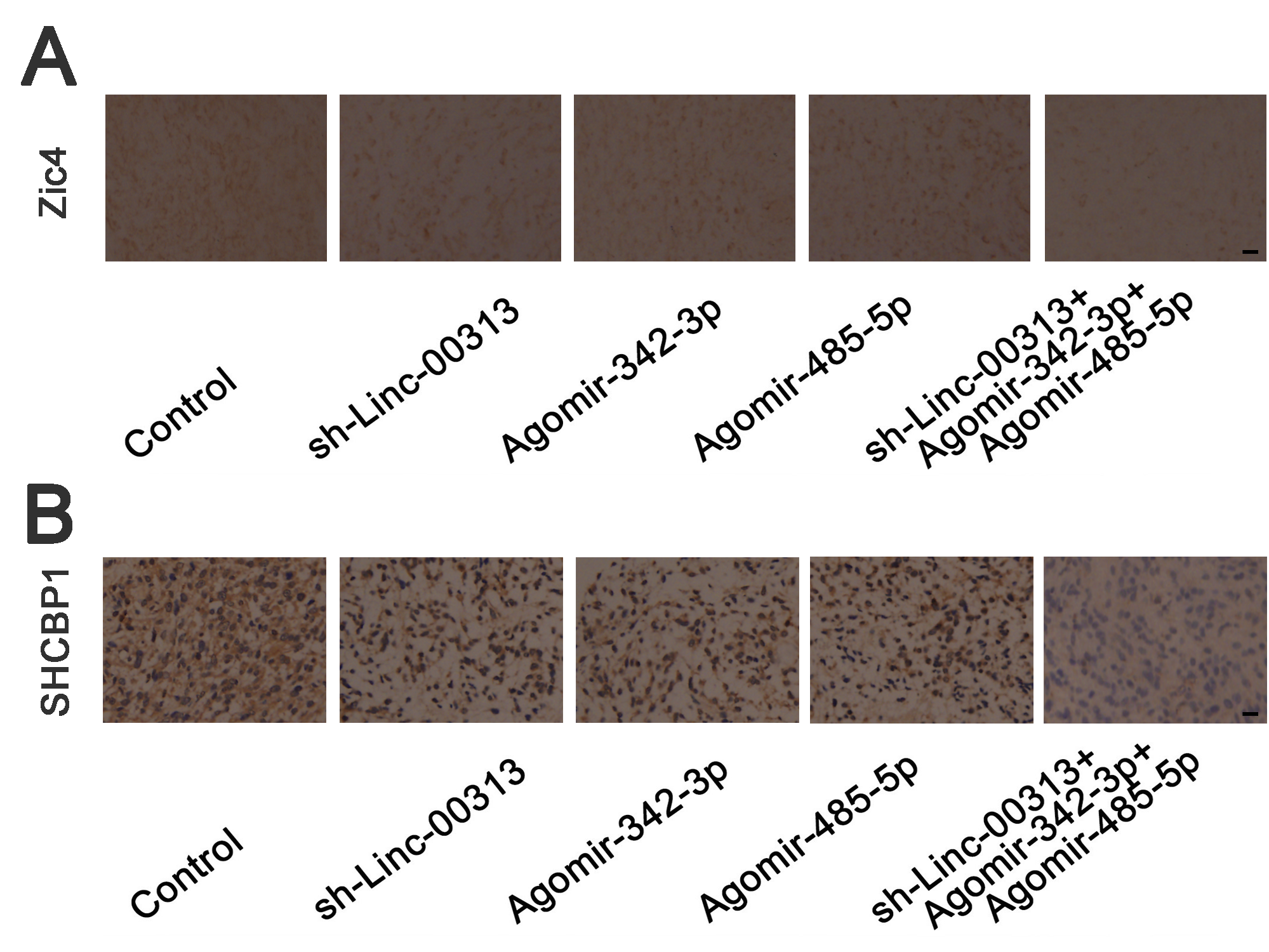

Supplement: Supplementary file 7 — Figure-S5 [file 41419_2019_1845_MOESM7_ESM.tif]

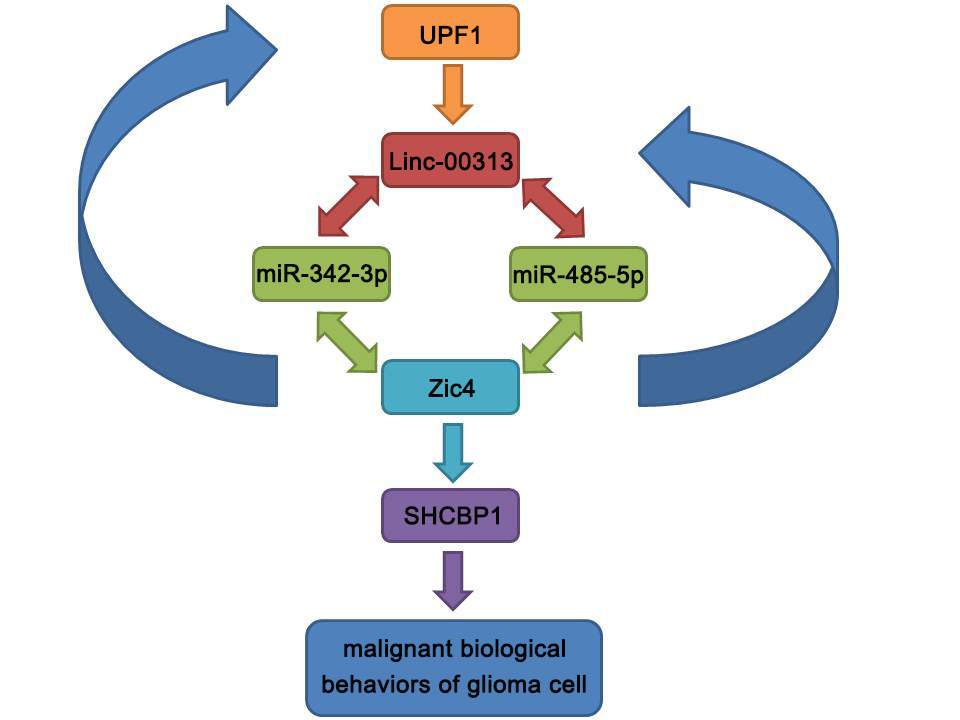

Supplement: Supplementary file 9 — Figure-S7 [file 41419_2019_1845_MOESM9_ESM.tif]

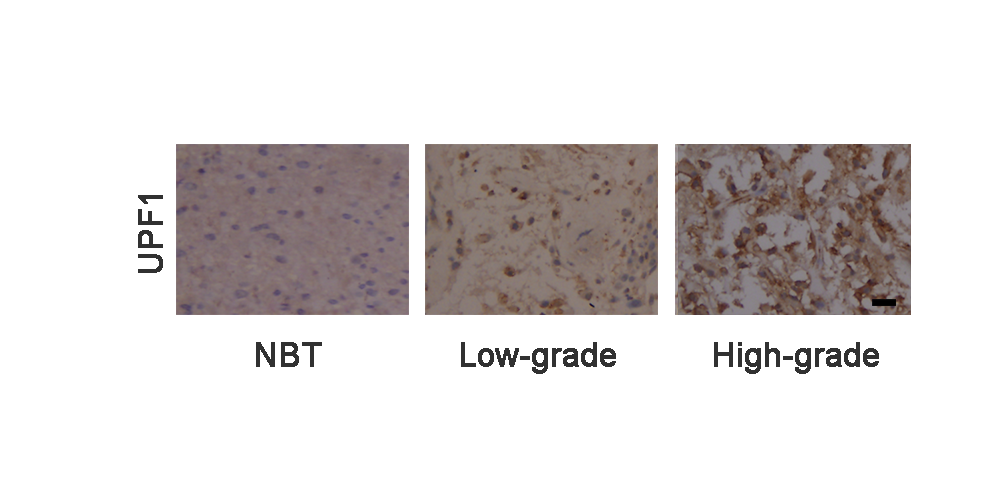

Supplement: Supplementary file 11 — Figure-S9 [file 41419_2019_1845_MOESM11_ESM.tif]

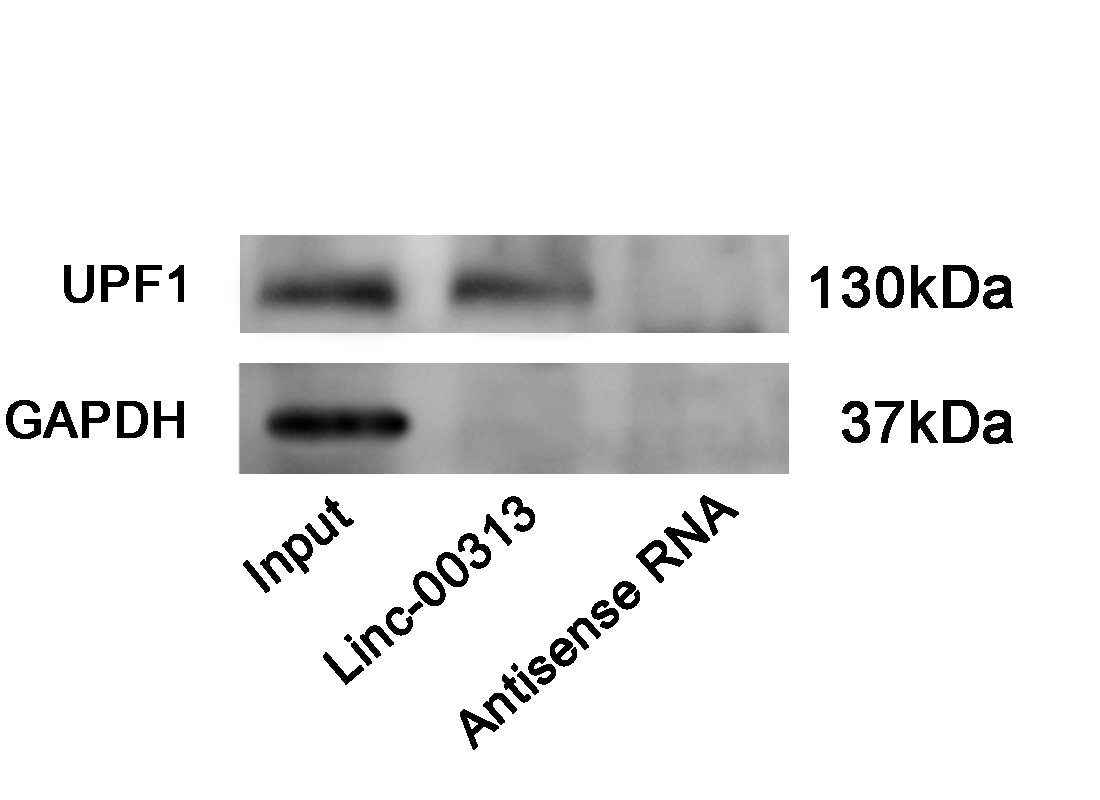

Supplement: Supplementary file 13 — Figure-S11 [file 41419_2019_1845_MOESM13_ESM.tif]

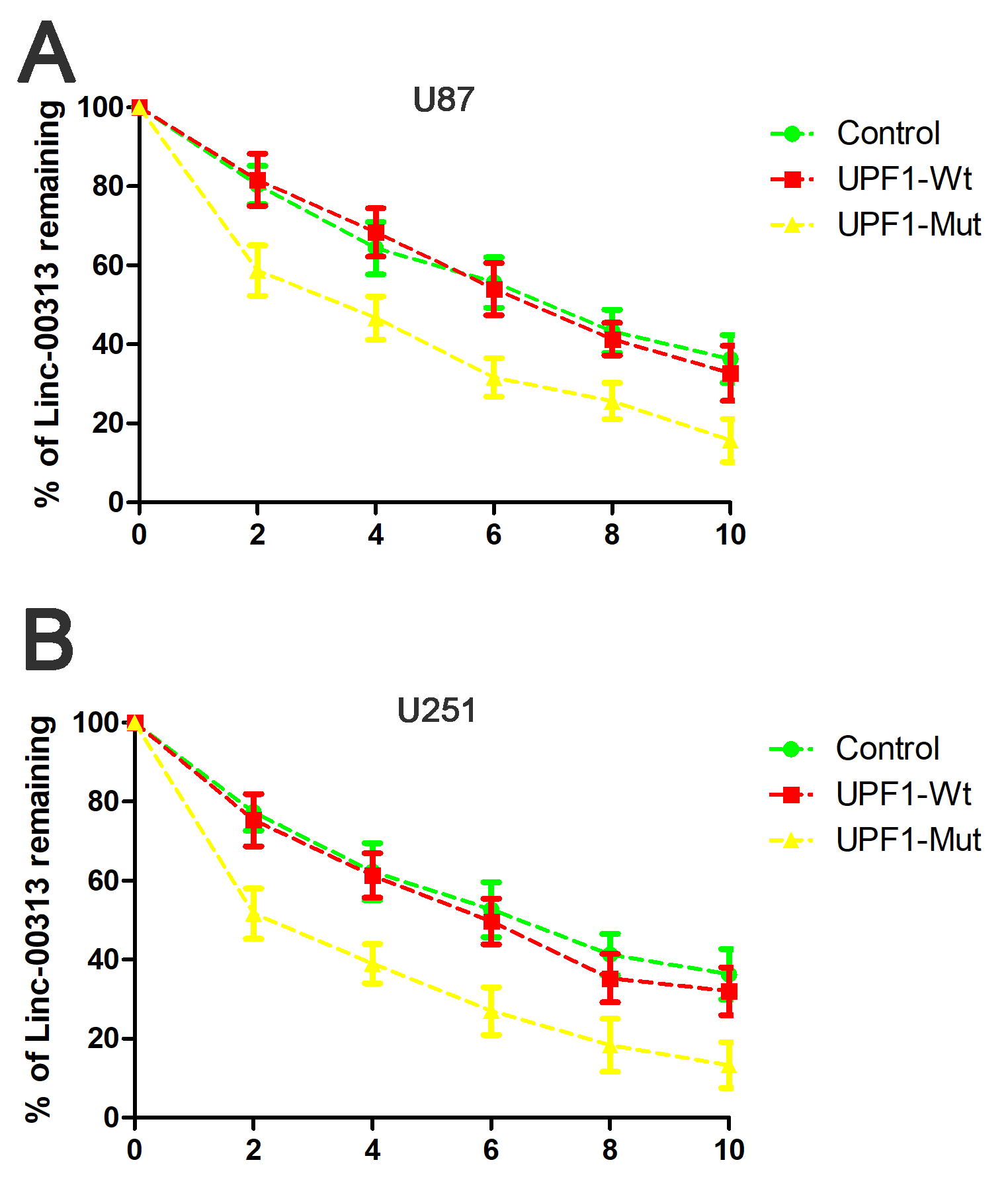

Supplement: Supplementary file 14 — Figure-S12 [file 41419_2019_1845_MOESM14_ESM.tif]

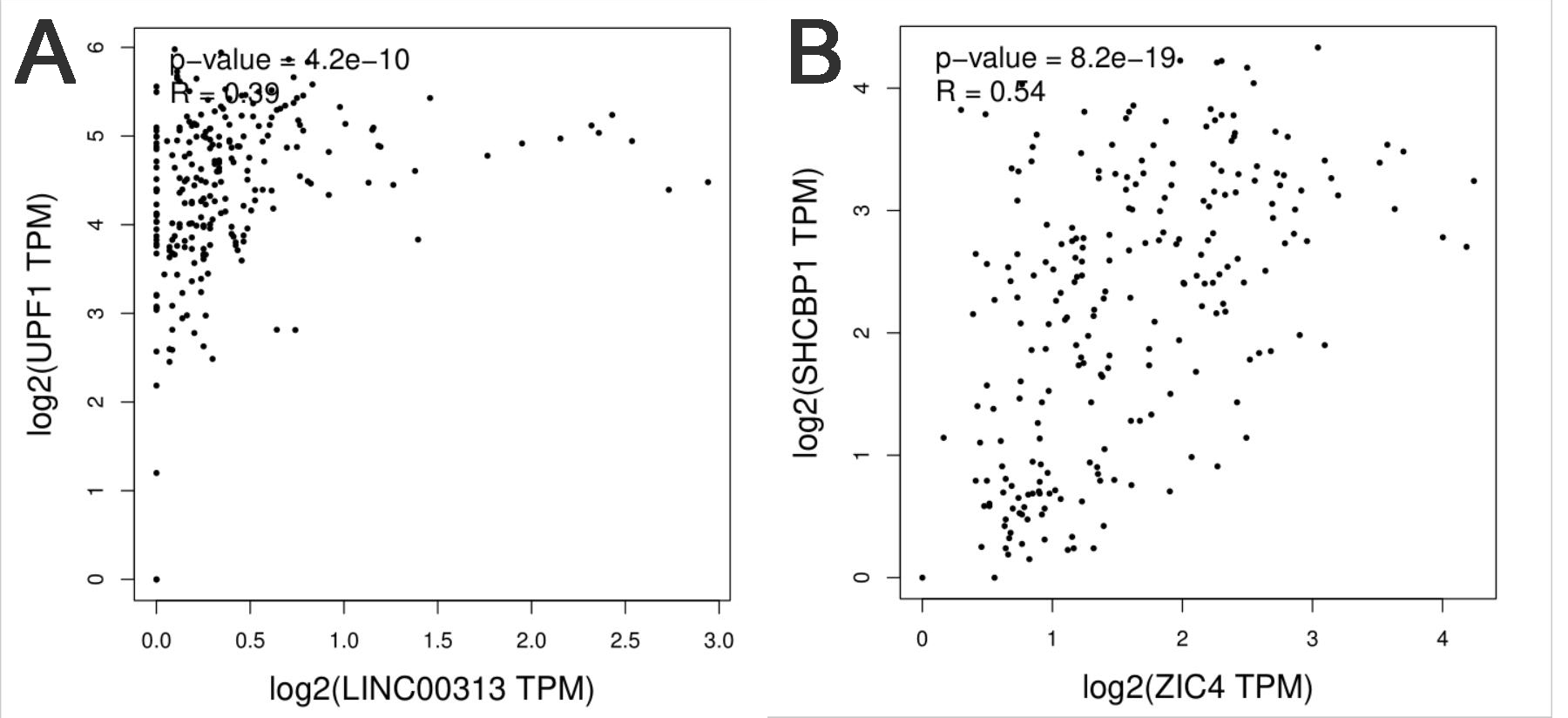

Supplement: Supplementary file 15 — Figure-S13 [file 41419_2019_1845_MOESM15_ESM.tif]
